# LncRNA *Airn* maintains LSEC differentiation to alleviate liver fibrosis via the KLF2-eNOS-sGC pathway

**DOI:** 10.1186/s12916-022-02523-w

**Published:** 2022-09-29

**Authors:** Ting Chen, Zhemin Shi, Yanmian Zhao, Xiaoxiang Meng, Sicong Zhao, Lina Zheng, Xiaohui Han, Zhimei Hu, Qingbin Yao, Huajiang Lin, Xiaoxiao Du, Kun Zhang, Tao Han, Wei Hong

**Affiliations:** 1 Department of Histology and Embryology, School of Basic Medical Sciences, Tianjin Medical University, Tianjin, China; 2 Department of Hepatology and Gastroenterology, Tianjin Union Medical Center, Tianjin Medical University, Tianjin Union Medical Center affiliated to Nankai University, Tianjin, China

**Keywords:** Liver fibrosis, lncRNA, LSECs, KLF2, eNOS-sGC

## Abstract

**Background:**

Long noncoding RNAs (lncRNAs) have emerged as important regulators in a variety of human diseases. The dysregulation of liver sinusoidal endothelial cell (LSEC) phenotype is a critical early event in the fibrotic process. However, the biological function of lncRNAs in LSEC still remains unclear.

**Methods:**

The expression level of lncRNA *Airn* was evaluated in both human fibrotic livers and serums, as well as mouse fibrotic livers. Gain- and loss-of-function experiments were performed to detect the effect of *Airn* on LSEC differentiation and hepatic stellate cell (HSC) activation in liver fibrosis. Furthermore, RIP, RNA pull-down-immunoblotting, and ChIP experiments were performed to explore the underlying mechanisms of *Airn*.

**Results:**

We have identified *Airn* was significantly upregulated in liver tissues and LSEC of carbon tetrachloride (CCl_4_)-induced liver fibrosis mouse model. Moreover, the expression of *AIRN* in fibrotic human liver tissues and serums was remarkably increased compared with healthy controls. In vivo studies showed that *Airn* deficiency aggravated CCl_4_- and bile duct ligation (BDL)-induced liver fibrosis, while *Airn* over-expression by AAV8 alleviated CCl_4_-induced liver fibrosis. Furthermore, we revealed that *Airn* maintained LSEC differentiation in vivo and in vitro. Additionally, *Airn* inhibited HSC activation indirectly by regulating LSEC differentiation and promoted hepatocyte (HC) proliferation by increasing paracrine secretion of Wnt2a and HGF from LSEC. Mechanistically, *Airn* interacted with EZH2 to maintain LSEC differentiation through KLF2-eNOS-sGC pathway, thereby maintaining HSC quiescence and promoting HC proliferation.

**Conclusions:**

Our work identified that *Airn* is beneficial to liver fibrosis by maintaining LSEC differentiation and might be a serum biomarker for liver fibrogenesis.

**Supplementary Information:**

The online version contains supplementary material available at 10.1186/s12916-022-02523-w.

## Background

Liver fibrosis is a dynamic wound-healing response to the continuous action of various injury factors, including non-alcoholic steatohepatitis, alcohol abuse, biliary obstruction, hepatitis B and C, and several other etiologies [1, 2]. It is characterized by excessive accumulation of extracellular matrix components and can eventually lead to cirrhosis and even hepatocellular carcinoma (HCC) [2, 3]. Thus, it is clear that there is an urgent need to develop novel diagnostic and therapeutic strategies for early stage of liver fibrosis.

LSEC are highly specialized endothelial cells located between blood cells and hepatocyte, facilitating the steric transport of cargo from the sinusoidal space to the space of Disse and into the parenchyma [4, 5]. Under physiological conditions, the vital characterizations of LSEC are fenestrated, absence of diaphragm, and lack of basement membrane. Meanwhile, it can serve to maintain HSC quiescence [6] and have the potential to promote HC regeneration [7]. Under fibrotic conditions, LSEC lose fenestrations and form a continuous basement membrane. This phenomenon is called “capillarization,” which precedes the activation of HSC and the onset of liver fibrosis, suggesting that it could be a preliminary step necessary for fibrogenesis [8]. Therefore, targeting LSEC might be a therapeutic approach to reverse liver fibrosis. Noteworthy, the fenestrated LSECs is maintained by two pathways: the eNOS-sGC pathway and NO-independent pathway [8]. It has been reported that the eNOS activity is impaired in LSEC of fibrotic liver, while sGC activator can rescue the eNOS activity and restore LSEC fenestration [9]. Additionally, KLF2, a transcriptional factor, upregulates eNOS expression and is essential to maintain functional endothelial phenotype [10, 11]. Furthermore, increasing KLF2 in cirrhotic animals improves LSEC phenotype, ameliorates the dysfunctional endothelium, reduces oxidative stress, and deactivates HSC, thereby turning into regression of cirrhosis [11]. Hence, further understanding of the cellular and molecular mechanism of LSEC may contribute to the development of more effective treatments.

LncRNAs are transcripts longer than 200 nucleotides but without protein coding potential [12]. Thus far, accumulating evidences demonstrated that lncRNAs participate in diverse physiological and pathological processes and play critical regulatory roles in human diseases [13, 14]. LncRNA *Airn* (Antisense Igf2r RNA) is an imprinted and paternal expressed gene, which is nuclear localized and highly unstable in the form of non-splicing 108 kb ncRNA, whereas the spliced *Airn* isoforms are as stable as other RNAs and are exported into the cytoplasm [15]. Furthermore, Hosen et al. demonstrated that *Airn* silencing reduced translation of IGF2BP2 protein and caused less binding of IGF2BP2 to target genes involved in cell survival, thereby augmenting cell death and reducing cell migration in cardiomyocytic HL-1 cells [16]. However, the role and the underlying mechanism of *Airn* in the development of liver fibrosis remain largely unclear.

In the present study, we aimed to elucidate the role of *Airn* in liver fibrosis. The results showed that *Airn* was significantly upregulated in liver tissues and LSEC of CCl_4_-induced liver fibrosis mouse model. Moreover, the expression of *AIRN* in livers and serums of live fibrosis patients was remarkably increased compared with healthy controls. In vivo studies revealed that *Airn* over-expression alleviated liver fibrosis. Additionally, it demonstrated that *Airn* maintained LSEC differentiation in vivo and in vitro through the KLF2-eNOS-sGC pathway, thereby suppressing HSC activation and promoting HC proliferation. Altogether, our results indicated that *Airn* was a critical and novel regulator of LSEC in liver fibrosis.

## Methods

### Study population

Study population analysis was performed as described in the previous study [17]. Briefly, 28 human fibrotic livers and 6 human healthy livers from patients with hepatic hemangioma were obtained from surgical resections without preoperative treatment at Tianjin Third Central Hospital (Tianjin, China). Hepatic fibrosis was scored (stages F0–F4) according to the METAVIR fibrosis staging system by three hepatopathologists blinded to the study protocol and stratified as normal liver (F0), mild fibrosis (F1–F2), and advanced fibrosis (F3–F4). In addition, we collected 47 serum samples from patients diagnosed as cirrhosis at Tianjin Third Central Hospital (Tianjin, China), and 30 matched blood donor volunteers recruited from the same hospital with no medical history. All subjects were of the same ethnicity. Clinical and pathological characteristics including age, gender, ALT, AST, ALB, GTT, and etiologies were recorded and summarized in Additional file 1: Table S1. The study has been approved by the local Ethical Committee of Tianjin Third Central Hospital (Tianjin, China). Written informed consent was obtained from each patient according to the policies of the committee. The study methodologies were conformed to the standards set by the Declaration of Helsinki.

### Animal in vivo study

All the animal protocols were in accordance with the Guidelines for Animal Experiments of Tianjin Medical University Animal Care and Use Committee. *Airn* knockout (*Airn*-KO) C57BL/6N mice were generated by CRISPR/Cas9 system (Cyagen, Suzhou China). In brief, genomic DNA was extracted from the *Airn*-KO mice and was PCR amplified using the primers (F: 5′-AGACACATTTAGTTGGTGGTTGGTCG-3′, R: 5′-TCTTCCACACCCAGGTGGCTTT-3′, R: 5′-AGGAAGTAGGCTCATGGGAGGAG-3′). The product of 800 bp was used for the amplicon and the sequence was confirmed by Sanger sequencing. All wild type (WT) and *Airn*-KO mice were generated from *Airn* heterozygous mice, they were kept in a standard 12-h light–dark cycle under the specific pathogen-free conditions with free access to water and food. All liver fibrosis models were performed in male mice unless indicated. For CCl_4_-induced liver fibrosis model, twenty WT mice and twenty *Airn*-KO mice were randomly divided into four groups: WT mice intraperitoneally injected oil (WT, *n* = 10), WT mice intraperitoneally injected CCl_4_ (WT + CCl_4_, *n* = 10), *Airn*-KO mice intraperitoneally injected oil (*Airn*-KO, *n* = 10), and *Airn*-KO mice intraperitoneally injected CCl_4_ (*Airn*-KO + CCl_4_, *n* = 10). They were administered 5% CCl_4_ (v/v) (Sigma-Aldrich, St. Louis, MO, USA) dissolved in oil (0.3 ml/kg body weight) thrice per week for 6 weeks via intraperitoneal injection. For BDL-induced liver fibrosis model, forty WT mice and forty *Airn*-KO mice were randomly divided into four groups. WT mice were treated with sham operation (WT, *n* = 20), WT mice were treated with BDL (WT + BDL, *n* = 20), *Airn*-KO mice were treated with sham operation (*Airn*-KO, *n* = 20) and *Airn*-KO mice were treated with BDL (*Airn*-KO + BDL, *n* = 20). Eighteen days later, all mice were sacrificed under anesthesia with 3% sodium pentobarbital (45 mg/kg, ip). For over-expressed *Airn* model, adeno-associated virus (AAV8) vectors were used to over-express *Airn* in mice, and AAV8-*GFP* was used as a control. Thus, forty Balb/c mice were divided into four groups randomly: Mice were treated with oil in combination with injection of AAV8-*GFP* (AAV8-*GFP*, *n* = 10), CCl_4_ in combination with injection of AAV8-*GFP* (AAV8-*GFP* + CCl_4_, *n* = 10), oil in combination with injection of AAV8-*Airn* (AAV8-*Airn*, *n* = 10), and CCl_4_ in combination with injection of AAV8-*Airn* (AAV8-*Airn* + CCl_4_, *n* = 10). Mice in AAV8-*GFP* + CCl_4_ group and AAV8-*Airn* + CCl_4_ group were injected with AAV8-*GFP* and AAV8-*Airn* respectively via the tail vein 2 weeks after the first injection of CCl_4_, and administered 5% CCl_4_ (v/v) dissolved in oil (0.2 ml/kg body weight) thrice per week via intraperitoneal injection. AAV8-*GFP* and AAV8-*Airn* group animals were injected with an equivalent volume of oil. After treatment with CCl_4_ for 8 weeks, all mice were sacrificed under anesthesia with 3% sodium pentobarbital (45 mg/kg, ip).

### Histology and immunohistochemistry

The immunohistochemistry was performed essentially as described previously [18]. The slides were treated with primary antibody α-SMA (1:200, rabbit polyclonal, Abcam, ab5694), COL1α1 (1:200, rabbit polyclonal, Abcam, ab34710), CD31 (1:25, mouse monoclonal, ab9498), LAMININ (1:200, rabbit polyclonal, Abcam, ab11575), and PCNA (1:800, rabbit monoclonal, Cell Signaling Technology, #13110), overnight at 4 °C. The slides were incubated with secondary antibody (1:500) (HRP-conjugated anti-rabbit IgG), and the reaction products were visualized using diaminobenzidine (DAB) and monitored by microscopy.

### Scanning electron microscopy

Briefly, livers were fixed with glutaraldehyde, postfixed with OsO4, dehydrated with graded alcohols, dried with hexamethyldisilazane, sputter-coated with gold, and examined using a Gemini 300 scanning electron microscope (Zeiss, Germany). Porosity (percentage of LSEC surface occupied by fenestrae) was measured in scanning electron microscopy (SEM) micrographs of cells.

### Isolation and culture of primary HCs, HSCs, KCs, and LSECs

Primary mouse HSC and HC were isolated by pronase/collagenase perfusion digestion followed by density gradient centrifugation, as previously described [17]. In brief, primary LSEC were isolated from the 8-week-old Balb/c mice by in situ perfusion with 30 ml SC1 solution and 30 ml 0.05% Collagenase IV solution sequentially. The cell suspension was centrifuged at 50*g* for 4 min and then the supernatant was centrifuged at 500*g* for 8 min at 4 °C. Pelleted cells were resuspended in 10 ml of 18% Nycodenz solution (Sigma-Aldrich, St. Louis, MO, USA); 6 ml of 12% Nycodenz solution, 6 ml of 8% Nycodenz, and 4 ml of DMEM were orderly loaded on the top of the cell suspension. The added gradient centrifugal liquid was centrifuged at 1450*g* and 4 °C for 22 min without brake. LSECs were recovered from the interface between the 8 and 12% Nycodenz solutions, mixed with an equal volume of DMEM and centrifuged at 600*g* for 6 min at 4 °C. Cells were resuspended and incubated with anti-CD146 magnetic beads (Miltenyi Biotec, Bergisch Gladbach, Germany). Finally, LSEC were cultured in collagen IV-coated plates with LSEC medium, and cell viability was determined by the trypan blue exclusion method.

### RNA pull-down

*Airn* was transcribed in vitro with T7 RNA polymerase (Thermo Scientific #K0441, MA, USA), and it was further treated with RNase-free DNase I to remove excess DNA. Next, the RNA was purified, biotin labeled (Thermo Scientific 20163, MA, USA), and attached with streptavidin agarose bead (Thermo Scientific 20164, MA, USA). Whole-cell lysates from freshly liver cell suspension were added to the labeled RNA according to the manuscript. The recruited proteins were subjected to western blot analysis.

### Chromatin immunoprecipitation (ChIP)

ChIP assays were performed essentially as described previously [18, 19]. Anti-EZH2 (rabbit polyclonal, Abcam, ab186006) and IgG were used to immunoprecipitated chromatin fragments. Finally, qRT-PCR assays were performed to analyze the precipitated chromatin DNA. The immunoprecipitated DNA was quantitated by qRT-PCR. The ChIP primer sequences were as follows: Klf2-pro 1(-272--120) (Forward) 5′-GCGCGCTAACTATGCTGTTG-3′ and Klf2- pro 1 (Reverse) 5′-CGGTATATAAGCCTGGCGGT-3′, Klf2-pro2 (-916--804) (Forward) 5′- GCTCCTTGGATGAGGC-TT-GT-3′ and Klf2-pro2 (Reverse) 5′-AGCATTAGGTTCAAGGCCCC-3′, Klf2- pro3 (-1299--1155) (Forward) 5′-TGTTTGCCTCCGGGGTTAAG-3′ and Klf2- pro3 (Reverse) 5′-GGGGGATGGGCACATCAAAT-3′, Gapdh intron (Forward) 5′-ATCCTGTAGGCCAGGTGATG-3′ and (Reverse) 5′-AGGCTCAAGGGCTTTTAAGG-3′. The data of ChIP was shown as a percentage relative to input DNA.

### Plasmid constructs

The cDNA of full-length *Airn* was sequentially amplified by PCR and ligated into the lentiviral shuttle pCCL.PPT.hPGK.IRES.eGFP/pre [18] to generate the over-expression plasmid (LV-*Airn* and the empty plasmid as the LV-Control). The cloning primer sequences were as follows: LV-*Airn* BamHI Forward 5′- CGCGGATCCAATAATCTCCACCCCCTG-3′, LV-*Airn* BamHI Reverse 5′- CGCGGATCCTTAAGACCCTGTTGAAATTT-3′. These plasmids were used to produce lentivirus in HEK-293T cells with the packaging plasmids. Infectious lentiviruses were harvested at 36 and 60 h after transfection and filtered through 0.45 μm PVDF filters for in vitro experiments. AAV8 vectors for capsid screening were produced by transfecting AAV-293 cells using polyethylenimine (PEI) with an AAV vector plasmid (pAAV.CMV.PI.EGFP.WPRE.Bg-h, addgene, #105530) and helper plasmid including pAAV2/8 (addgene, #112864) and pAdDeltaF6 (addgene, #112867). For in vivo experiments, AAV vectors were produced in large scale and purified through iodixanol gradient density centrifugation, and full AAV particles were collected from the 40–60% interface of iodixanol phase for in vivo experiments. Male mice at 6–8-week-old were injected by tail vein with 1×10^12^ pfu/mouse genome copies of AAV8-GFP or AAV8-*Airn*. The cloning primer sequences were as follows: AAV8-*Airn* HindIII Forward 5′- CCCAAGCTTAATAATCTCCACCCCCTG-3′, AAV8-*Airn* BamHI Reverse 5′- CGCGGATCCTTAAGACCCTGTTGAAATTT-3′.

### Cell culture

The non-tumorigenic mouse hepatocyte cell line AML12 was cultured in DMEM with 10% fetal bovine serum (FBS, Gibco, Gaithersburg, MD, USA), 1 × insulin-transferrin-sodium selenite media supplement (ITS, Sigma-Aldrich), 40 ng/ml dexamethasone, 100 U/ml penicillin, and 100 μg/ml streptomycin. The cell line HEK293T or AAV293 were maintained in DMEM supplemented with 10% fetal bovine serum, 100 U/ml penicillin, and 100 μg/ml streptomycin, both cells were cultured in humidified air at 37 °C and 5% CO_2_.

### Small interfering RNA (siRNA) transfection

Primary LSECs (1×10^6^/well) were seeded in collagen-coated 6-well plates and transfected with si*Airn*s and siRNA-Control for 48 h; RNA and protein of cells were harvested and isolated. si*Airn*s and siRNA-Control were gained from GenePharma, and the sequences are as follows: si*Airn*-1 (mouse), sense 5′-CCAGUUACCACGCAGACAUTT-3′ and antisense 5′-AUGUCUGCGUGGUAACUGGTT-3′, si*Airn*-2 (mouse), sense 5′- CCGUCACCAUGUGUCCUUUTT-3′ and antisense 5′- AAAGGACACAUGGUGACGGTT-3′, si*Airn*-3 (mouse), GCAGCUCUCAUCUGUGUUATT-3′ sense 5′- and antisense 5′- UAACACAGAUGAGAGCUGCTT-3′, NC (mouse), sense 5′-UUCUCCGAACGUGUCACGUTT-3′, and anti-sense 5′-ACGUGACACGUUCGGAGAATT-3′.

### Nuclear-cytoplasmic fractionation

Cytoplasmic and nuclear RNA and protein isolation were performed with PARIS™ Kit (Invitrogen, Grand Island, NY, USA), following the manufacturer’s instruction and were performed essentially, as described previously [18].

### RNA-seq and computational analysis

Briefly, primary LSECs infected with two separated si*Airn* were lysed with Trizol reagent. Total RNA was qualified and quantified using a Nano Drop and Agilent 2100 bioanalyzer (Thermo Fisher Scientific, MA, USA). The library was amplified with phi29 to make DNA nanoball (DNB) which had more than 300 copies of one molecule, DNBs were loaded into the patterned nanoarray, and single-end 50 bases reads were generated on BGIseq500 platform (BGI-Shenzhen, China). The threshold we used to screen up- or downregulated mRNAs was fold change >1.4 and *p*<0.05. The transcriptome sequencing data have been deposited in NCBI Gene Expression Omnibus (GEO) under the following accession number: GSE174175.

### Fluorescence in situ hybridization (FISH)

*Airn* probes were synthesized by GenePharma Technology (Shanghai, China). FISH was performed using a FISH Kit (GenePharma) according to the manufacturer’s instructions. Nuclei were stained with DAPI. Images were acquired on a Zeiss confocal microscope LSM700. The sequences of FISH probe were as follows: probe1: 5′- TATAATGTTGAAGCCTCGGC-3′, probe 2: 5′-CAGGATGTCTGCGTGGTAAC-3′, probe 3: 5′-ATTTCTAAGGTGGTTTCCGA -3′, probe 4: 5′-TCTGTAGTTTTCTAATGGCC-3′, probe 5: 5′-CTGGGGAAAGAAGTGTGTCT-3′, probe 6: 5′-TTTTTTTAAGACCCTGTTGA-3′.

### Western blot analysis

Cells were lysed with cell lysis buffer (Cell Signaling Technology) supplemented with protease inhibitor cocktail, 1% PMSF, and 1% phosphatase inhibitor. Protein concentrations were measured by the BCATM Protein Assay Kit (Bio-Rad Laboratories, Hercules, CA, USA) using BSA as standard. Appropriate amount of protein samples (40 μg for liver tissues; 25–50 μg for cells) along with 4× loading buffer and ddH_2_O were boiled for 4 min and then subjected to sodium dodecyl sulfate–polyacrylamide gel electrophoresis. Following by electrophoresis, the separated proteins were blotted onto polyvinylidene fluoride (PVDF) membranes in transfer buffer with constant current of 300 mA for 3 h at 4 °C. Then the PVDF membranes were sequentially washed with TBST containing 0.2% Tween-20, blocked with 5% nonfat milk in TBST and incubated with the interested primary antibodies diluted in TBST containing 0.2% Tween-20 overnight at 4 °C. Antibodies used for immunoblotting included VEGFR2 (1:1000, rabbit monoclonal, Cell Signaling Technology, #9698), eNOS (1:1000, mouse monoclonal, Abcam, ab76198), VE-Cadherin (1:1000, rabbit polyclonal, Abcam, ab33168), PCNA (1:1000, rabbit monoclonal, Cell Signaling Technology, #13110), α-SMA (1:1000, rabbit polyclonal, Abcam, ab5694), COL1α1 (1:1000, rabbit polyclonal, Abcam, ab34710), MMP2 (1:1000, rabbit monoclonal, Abcam, ab92536), TIMP1 (1:1000, mouse monoclonal, Santa Cruz, sc-21734), KLF2 (1:1000, mouse polyclonal, Santa Cruz, sc-166238). GAPDH were severed as control for total protein amount. Then, all of membranes were incubated with the HRP-conjugated secondary antibody for 1 h at room temperature. Every experiment was repeated at least three times independently.

### Confocal microscopy

Immunofluorescence analysis was performed as described previously [20]. Antibodies used for confocal microscopy included VEGFR2 (1:200, rabbit monoclonal, Cell Signaling Technology, #9698), VE-Cadherin (1:200, rabbit polyclonal, Abcam, ab33168), F4/80 (1:50, rabbit monoclonal, Abcam, ab16911), α-SMA (1:200, rabbit polyclonal, Abcam, ab5694), COL1α1 (1:200, rabbit polyclonal, Abcam, ab34710), Ki67 (1:200, rabbit monoclonal, Abcam, ab16667), PCNA (1:800, rabbit monoclonal, Cell Signaling Technology, #13110), and KLF2 (1:50, mouse polyclonal, Santa Cruz, sc-166238). Cells were incubated with FITC-conjugated secondary antibodies (Thermo Fisher Scientific, HRP-conjugated anti-rabbit IgG or anti-mouse IgG, Alexa Fluor 594) in a dark environment at room temperature for 1 h. Next, cells away from light were stained with DAPI (Sigma, USA) for 20 min. Finally, the slides were mounted with an anti-fade mounting medium and were observed with a Zeiss confocal microscope LSM700.

### Quantitative real-time polymerase chain reaction

qRT-PCR analysis was performed as described previously [17]. The sequences of primers are listed as follows.

**Table:** 

Gene symbol	Forward 5′–3′	Reverse 5′–3′
*Gapdh* (mouse)	GGCATGGACTGTGGTCATGAG	TGCACCACCAACTGCTTAGC
*β-Actin* (mouse)	ATGCCACAGGATTCCATACCCAA	CTCTAGACTTCGAGCAGGAGATGG
*Airn* NR_002853.2	AAGCACAGCACCGCCAGT	CCATGTCCTTTCTTTTCCACTACC
*Airn* NR_027772.1	AAGCACAGCACCGCCAGT	CAAAGGTGCTTGCCTCCAA
*Airn* NR_027773.1	AAGCACAGCACCGCCAGT	CAGGACCTCAAGTCAGGAACCT
*Airn* NR_027784.1	AAGCACAGCACCGCCAGT	AGGCCTTTGTTCACATCTCTTCA
*eNos* (mouse)	GGCAACTTGAAGAGTGTGGG	CTGAGGGTGTCGTAGGTGATG
*Vegfr2* (mouse)	TTTGGCAAATACAACCCTTCAG	GCAGAAGATACTGTCACCACC
*VE-cadherin* (mouse)	CAGCACACTAGCCTGGTGTTA	CGCCCATGATTCTGCATGTAGA
*Lyve-1* (mouse)	CAGCACACTAGCCTGGTGTTA	CGCCCATGATTCTGCATGTAGA
*Cd31* (mouse)	ACCGGGTGCTGTTCTATAAGG	TCACCTCGTACTCAATCGTGG
*Et-1* (mouse)	CACCGTCCTCTTCGTTTTGC	GGCTCTGCACTCCATTCTCA
*Laminin* (mouse)	GAAAGGAAGACCCGAAGAAAA	CCATAGGGCTAGGACACCAAA
*Hgf* (mouse)	GCGAATTGGTGTTCTGCCTG	GAGATGCCGGGCTGAAAGAA
*Wnt2a* (mouse)	CTCGGTGGAATCTGGCTCTG	CACATTGTCACACATCACCCT
*Angpt2* (mouse)	AGAAGAGCAAACCACCTTCAG	GTCACAGTAGGCCTTGATCTCC
*Col1α1* (mouse)	ATCGGTCATGCTCTCTCCAAACA	ACTGCAACATGGAGACAGGTCAGA
*α-SMA* (mouse)	TCGGATACTTCAGCGTCAGGA	GTCCCAGACATCAGGGAGTAA
*Mmp2* (mouse)	GTGTTCTTCGCAGGGAATGAG	GATGCTTCCAAACTTCACGCT
*Pcna* (mouse)	TTTGAGGCACGCCTGATCC	GGAGACGTGAGACGAGTCCAT
*Ki67* (mouse)	CATCCATCAGCCGGAGTCA	TGTTTCGCAACTTTCGTTTGTG
*F4/80* (mouse)	TGACTCACCTTGTGGTCCTAA	CTTCCCAGAATCCAGTCTTT CC
*Cd11b* (mouse)	TCCTGTACCACTCATTGTGG	GGGCAGCTTCATTCATCATG
*GAPDH* (human)	ACCCAGAAGACTGTGGATGG	TTCAGCTCAGGGATGACCTT
*β-ACTIN* (human)	GCCGGGACCTGACTGACTAC	TTCTCCTTAATGTCACGCACGAT
*AIRN* NR_047514.1	GGAAAAGGGATGCGGTGTTT	CCTTTTCAGGACGTGACCCG
*AIRN* NR_047511.1	AACTTTTGCCCAGTCGGCTC	CCATGGCAGCTTGGTTTCAG

### Cell Counting Kit-8 (CCK-8) assay

Cell Counting Kit-8 (CCK-8) assay CCK-8 assay was carried out for detection of cell proliferation in AML12 cells. AML12 cells were inoculated in 96-well plates with 2×10^3^ cells per well. Absorbance at 450 nm was recorded at specified time points using the CCK-8 kit, based on which the viability curve was plotted.

### 5-Ethynyl-2’- deoxyuridine (EdU) assay

Cells were inoculated in 24-well plates (10^4^ cells/ well) and labeled with 10 μmol/L EdU at 37 °C for 2 h. After 15 min fixation in 4% paraformaldehyde, cells were incubated with phosphate buffered saline (PBS) containing 0.3% Triton-100 for 15 min. After washing with PBS, 125 μL of dying solution was applied per well and incubated in dark for 30 min. The nucleus was stained with 1×Hoechst 33342 for 10 min. EdU-positive cells, Hoechst-labeled nucleus, and merged ones were captured under a microscope.

### Statistical analysis

Data were expressed as mean ± SD. All the statistical analyses were performed with the SPSS 13.0 (IBM, Armonk, NY, USA). Statistical analyses were performed using either Student’s *t* test (two-group comparison) or one-way analysis of variance (more than two groups) followed by post hoc comparison, and differences with *p*<0.05 were considered significantly.

## Results

### Airn expression was upregulated in liver fibrosis

**Fig. 1:**
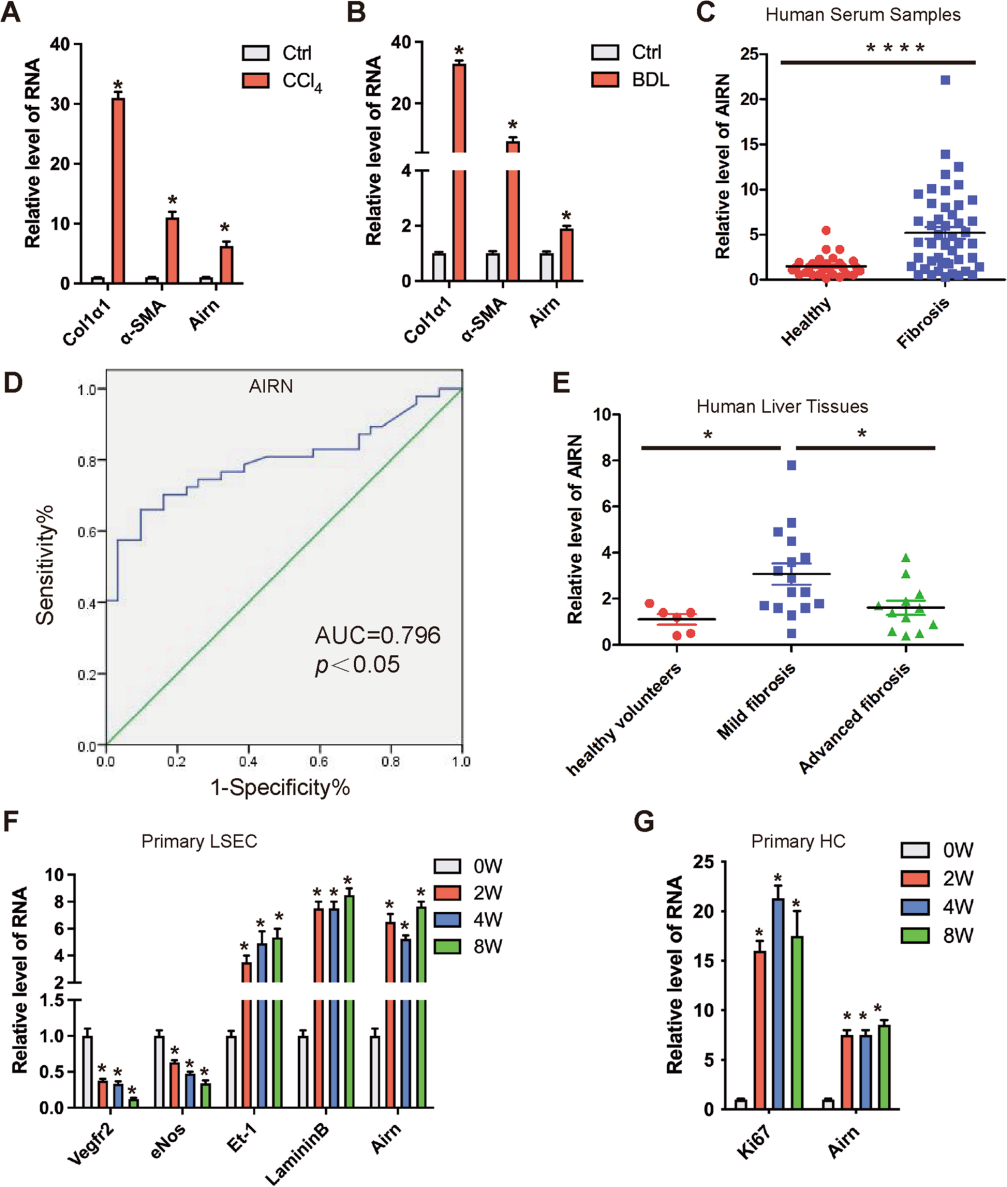
*Airn* expression was upregulated in liver fibrosis. **A, B** qRT-PCR analysis of *Col1α1*, *α-SMA*, and *Airn* in livers from mice treated with CCl_4_ or underwent BDL. **C** qRT-PCR analysis of *AIRN* in liver serum from healthy (*n* = 30) and cirrhosis serum (*n* = 47). **D** ROC curve analysis of *Airn* in the serums of liver fibrosis patients and healthy individuals. AUC = area under the curve. **E** qRT-PCR analysis of *AIRN* in liver samples from healthy (*n* = 6), mild fibrosis (*n* = 16), and advanced fibrosis (*n* = 12) patients. **F** Primary LSEC were isolated from livers of mice treated with CCl_4_ or oil for 0, 2, 4, and 8 weeks and the transcript of *Vegfr2*, *eNos*, *Et-1*, *Laminin*, and *Airn* were determined by qRT-PCR. **G** Primary HC were isolated from livers of mice treated with CCl_4_ or oil for 0, 2, 4, and 8 weeks. The expression of *Ki67* and *Airn* was detected by qRT-PCR. The data are expressed as the mean ± SD for at least triplicate experiments, *p< 0.05

In our prior study, we found that *Airn* was significantly upregulated in liver fibrosis according to the microarray data. (The microarray data discussed in the previous article have been deposited in NCBI Gene Expression Omnibus and are accessible through GEO Series accession number GSE89147) [18]. As both mouse and human *Airn* have various isoforms, we first detected these *Airn* isoforms in liver tissues or hepatocytes. Of all the isoforms, NR_002853 (mouse *Airn*) and NR_047514 (human *AIRN*) showed high expression in liver tissues and remarkably upregulated in fibrotic liver, compared with other isoforms (Additional file 1: Fig. S1A, B). To explore the role of *Airn* in liver fibrosis, the expression of *Airn* was initially verified in mouse liver fibrosis model. The results showed that the expression of *Airn* was dramatically increased in mouse CCl_4_- and BDL-induced fibrotic liver tissues compared with normal liver tissues (Fig. 1A, B). Moreover, total RNAs were isolated from the serums of 47 patients with liver fibrosis and found that the level of *AIRN* was increased in these patients compared with 30 healthy volunteers (Fig. 1C). ROC curve analysis revealed the potential diagnostic performance of *AIRN* in discriminating patients with liver fibrosis from healthy subjects (AUC = 0.796; *p*<0.05) (Fig. 1D). In addition, the expression of human *AIRN* was also detected in 6 healthy liver tissues, 16 mild fibrotic liver tissues (F1–F2) and 12 advanced fibrotic liver tissues (F3-F4) [17]. As shown in Fig. 1E, the expression of *AIRN* was significantly upregulated in mild fibrotic livers but not in advanced fibrotic livers.

To assess the expression of *Airn* in different cell types of healthy and fibrotic livers, primary liver parenchymal cells and non-parenchymal cells, including HSC, LSEC, and KC, were isolated from livers of healthy and fibrotic mice respectively. The purity of primary liver non-parenchymal cells was examined by confocal microscopy detection of VE-Cadherin and VEGFR2 (LSEC marker) [21, 22], α-SMA (HSC marker), and F4/80 (KC marker) (Additional file 1: Fig. S1C). Meanwhile, qRT-PCR analysis was preformed to further detect the expression of *Lyve1*, *VE-cadherin*, *Vegfr2*, *F4/80*, *Cd11b*, and *α-SMA* (Additional file 1: Fig. S1D). Based on these observations, LSEC preparations appeared about 94% purity. Our data showed that the highest expression level of *Airn* was found in HSC and LSEC followed by HCs and KCs (Additional file 1: Fig. S2A). Moreover, the expression of *Airn* was upregulated in primary LSECs from fibrotic livers of mice treated with CCl_4_ for 2, 4, and 8 weeks (Fig. 1F), correlating with the reduction of expression of *Vegfr2* and *eNos*, yet correlating also with the enhancement of the expression of *endothelin-1*(*Et-1*) and *Laminin* (Fig. 1F). Moreover, *Airn* was increased in injured primary HC and activated HSC, correlating with the enhancement of the expression of *Ki67*, *Col1α1*, and *α-SMA* (Fig. 1G and Additional file 1: Fig. S2B). However, *Airn* expression had no significant difference in primary KCs of mice treated with CCl_4_ for various time periods (Additional file 1: Fig. S2C). Overall, the data showed an obvious enhancement of *Airn* in primary LSEC, HC, and HSCs at 2 weeks after CCl_4_ treatment, indicating *Airn* is involved in the initiation of fibrosis. The increased *Airn* in these cells maintaining a high level until significant fibrosis could be observed at 8 weeks of CCl_4_ treatment, together with the finding that *AIRN* was increased in human mild fibrotic livers, suggested that *Airn* plays a role in the progression of liver fibrosis.

### Airn deficiency aggravated CCl_4_- and BDL-induced liver fibrosis and LSEC capillarization in vivo

**Fig. 2:**
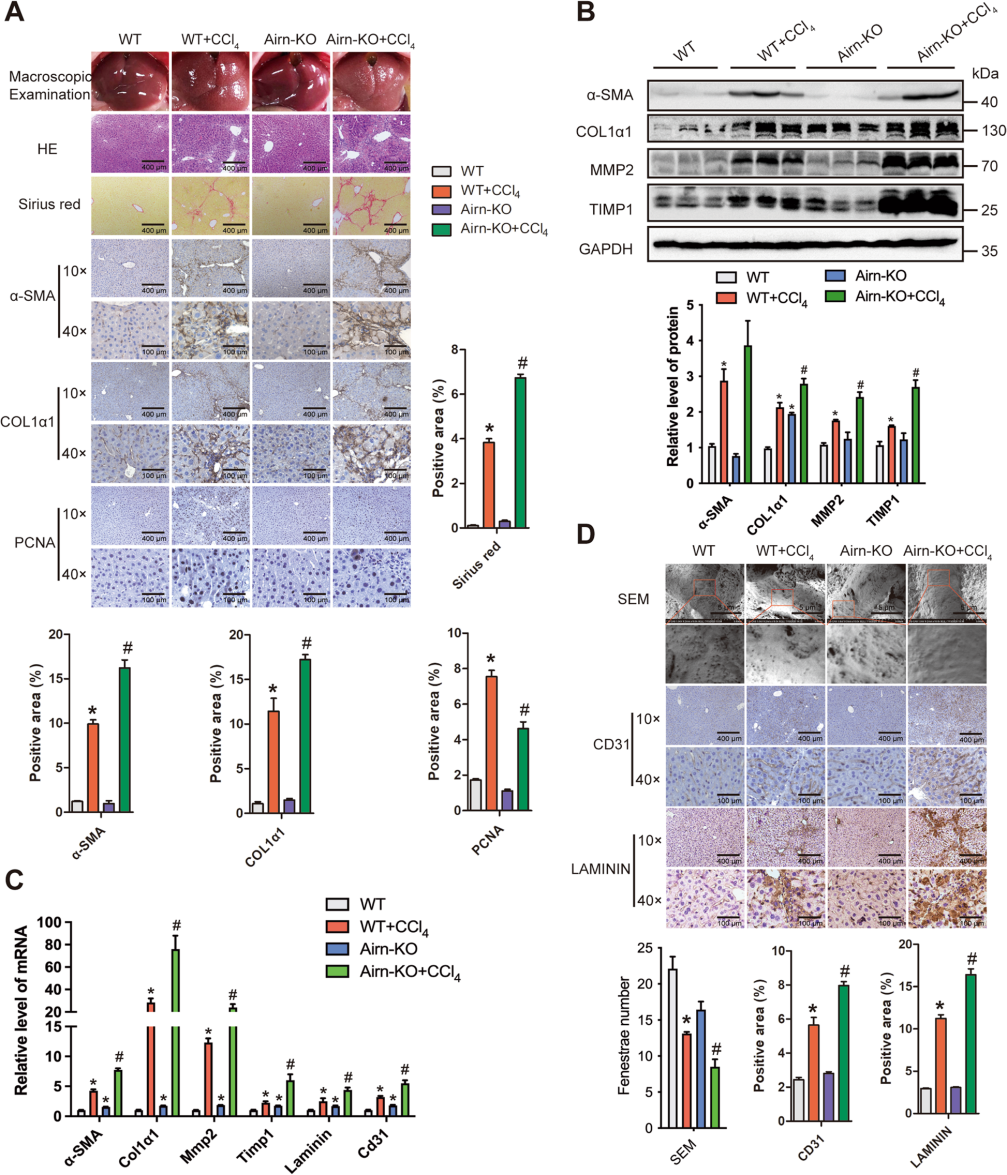
*Airn* deficiency aggravated CCl_4_-induced liver fibrosis and LSEC capillarization in vivo. C57BL/6 mice were divided into four groups: WT (*n* = 10), WT + CCl_4_ (*n* = 10), *Airn*-KO (*n* = 10), and *Airn*-KO +CCl_4_ (*n* = 10). **A** Liver fibrosis was evaluated by macroscopic examination, H&E staining, sirius red staining, and IHC for α-SMA, COL1α1, and PCNA; scale bar, 400 μm for 10× and 100 μm for 40×. Right, five images of each liver and five livers from different mice were quantified for each group. **B** The protein level of α-SMA, COL1α1, MMP2, and TIMP1 was determined by western blot and quantitatively compared with GAPDH as a reference control. **C** The mRNA level of the genes related to liver fibrosis including *α-SMA*, *Col1α1*, *Mmp2*, and *Timp1*, and LSEC capillarization markers *Cd31* and *Laminin* was determined by qRT-PCR. **D** Liver sections from CCl_4_-induced fibrotic liver were analyzed using SEM and the protein level of CD31 and LAMININ staining was detected by IHC and quantitatively compared; scale bar, 400 μm for 10× and 100 μm for 40×. Data were quantified from 10 random fields per group for SEM, *p<0.05 stands for WT + CCl_4_ or *Airn*-KO vs WT. #*p*<0.05 stands for *Airn*-KO + CCl_4_ vs WT + CCl_4_

In order to elucidate the function of *Airn* during liver fibrogenesis in vivo, we generated *Airn* knockout (*Airn*-KO) mice via CRISPR/Cas9 system (Additional file 1: Fig. S3A-D) and subsequently constructed the CCl_4_-induced liver fibrosis model. *Airn*-KO mice were normal in appearance and mating, and a detailed histological examination of major internal organs did not reveal any morphological abnormalities (Additional file 1: Fig. S4). The CCl_4_ group mice developed serious liver fibrosis and knockout of *Airn* further aggravated the CCl_4_-induced liver fibrosis as demonstrated by macroscopic examination, hematoxylin and eosin (H&E), sirius red staining, serum ALT, AST level, and liver hydroxyproline content (Fig. 2A and Additional file 1: Table S2). Moreover, knockout of *Airn* notably facilitated the upregulation of CCl_4_-induced α-SMA and COL1α1, whereas it significantly suppressed compensatory upregulation of CCl_4_-induced PCNA by IHC (Fig. 2A). In addition, western blot and qRT-PCR analysis showed that deficiency of *Airn* significantly promoted the upregulation of CCl_4_-induced α-SMA, COL1α1, MMP2, and TIMP1 (Fig. 2B, C), suggesting *Airn* deficiency aggravated CCl_4_-induced liver fibrosis. On the other hand, we investigated whether *Airn* regulated LSEC differentiation or capillarization in vivo. Scanning electron microscopy (SEM) results showed that the number of fenestrae were noticeably decreased in CCl_4_-induced mice and were further decreased in CCl_4_-induced *Airn*-KO mice (Fig. 2D). Furthermore, the expression of the genes related to LSEC capillarization including CD31 and LAMININ was observably enhanced in CCl_4_-induced *Airn*-KO mice when compared with CCl_4_-induced WT mice (Fig. 2C, D), suggesting that *Airn* deletion worsened CCl_4_-induced LSEC capillarization.

To exclude the possibility that *Airn* alters the metabolism or toxicity of CCl_4_ rather than by altering cell responses, the results was confirmed in a BDL-induced mouse liver fibrosis model. As shown in Additional file 1: Fig. S5A-C and Additional file 1: Table S3, the mice of BDL group developed serious liver fibrosis, and knockout of *Airn* further aggravated BDL-induced liver fibrosis as demonstrated by macroscopic examination, H&E staining, sirius red staining, IHC, serum ALT, AST level, liver hydroxyproline content, western blot, and qRT-PCR. As expected, knockout of *Airn* notably facilitated the upregulation of BDL-induced expression of LSEC capillarization marker genes CD31 and LAMININ assessed qRT-PCR and IHC (Additional file 1: Fig. S5C, D). Taken together, our data clearly revealed that *Airn* deficiency aggravated CCl_4_- and BDL-induced liver fibrosis and LSEC capillarization in vivo.

### Over-expression of Airn alleviated CCl_4_-induced liver fibrosis in vivo

**Fig. 3:**
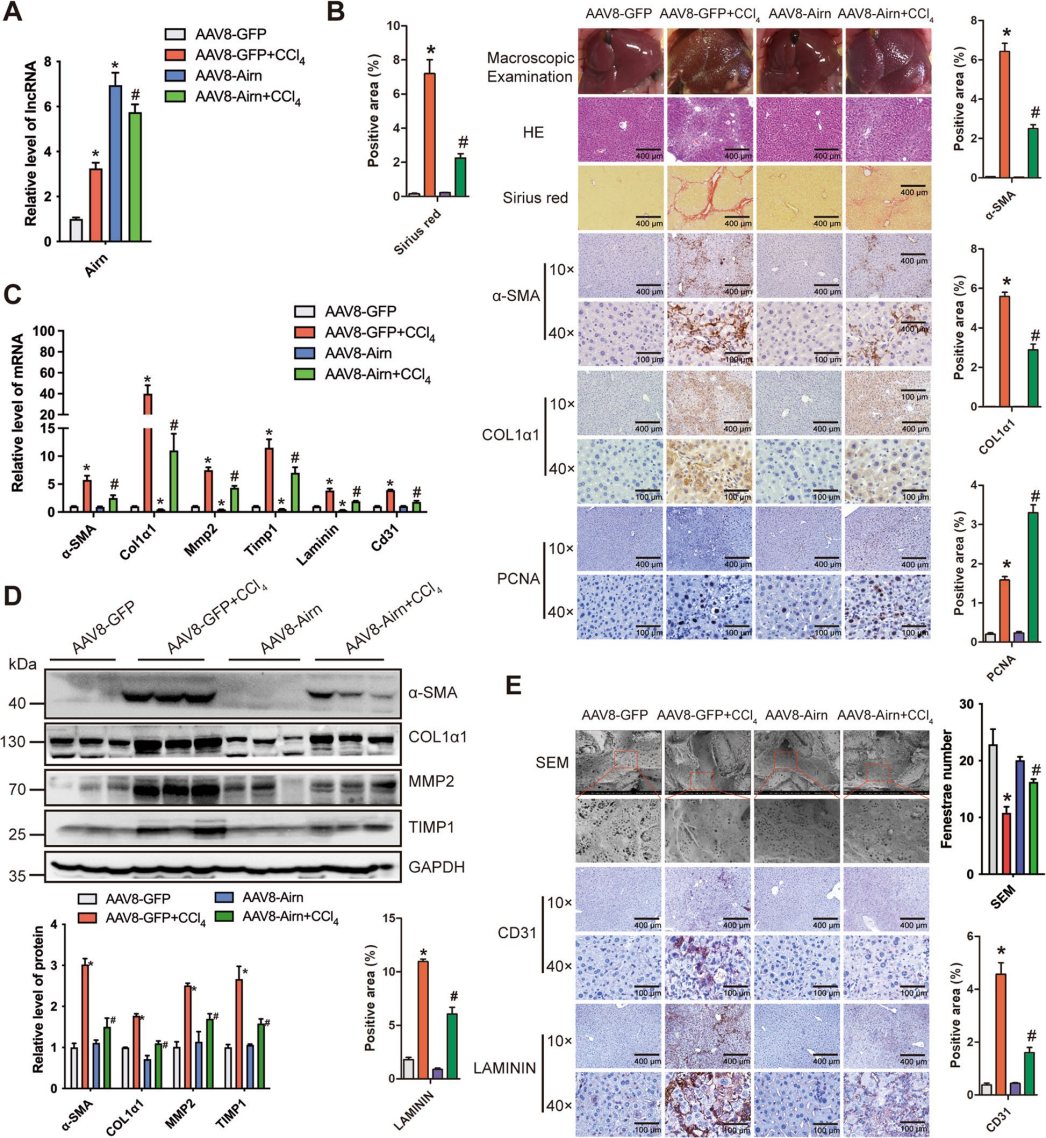
Over-expression of *Airn* alleviated CCl_4_-induced liver fibrosis in vivo*.* Mice were divided into four groups: AAV8-GFP (*n* = 10), AAV8-GFP + CCl_4_ (*n* = 10), AAV8-*Airn* (*n* = 10), and AAV8-*Airn* + CCl_4_ (*n* = 10), and transduced with AAV8-GFP or AAV8-*Airn* virus via tail vein 2 weeks after the first injection of CCl_4_, after 8 weeks of CCl_4_ treatment. **A** The expression of *Airn* in livers of each group was examined by qRT-PCR. **B** Liver fibrosis was evaluated by macroscopic examination, H&E staining, sirius red staining, and IHC for α-SMA, COL1α1, and PCNA; scale bar, 400 μm for 10× and 100 μm for 40×. Right, five images of each liver and five livers from different mice were quantified for each group. **C** The protein level of α-SMA, COL1α1, MMP2, and TIMP1 was determined by western blot and quantitatively compared with GAPDH as a reference control. **D** The mRNA level of *α-SMA*, *Col1α1*, *Mmp2*, and *Timp1*, and *Cd31* and *Laminin* was determined by qRT-PCR. **E** Liver sections from CCl_4_-induced fibrotic liver were analyzed using SEM and the protein level of CD31 and LAMININ was detected by IHC staining and quantitatively compared; scale bar, 400 μm for 10× and 100 μm for 40×. *p<0.05 stands for AAV8-*GFP*+CCl_4_ or AAV8-*Airn* vs AAV8-*GFP*. #*p*<0.05 stands for AAV8-*Airn*+CCl_4_ vs AAV8-*GFP*+CCl_4_

To test whether over-expression of *Airn* would alleviate liver fibrosis in vivo, AAV8-*Airn* or AAV8-*GFP* was intravenously injected into the CCl_4_-treated or oil-treated mice via the tail vein 2 weeks after the first injection of CCl_4_. After 8 weeks of CCl_4_ treatment, qRT-PCR analysis confirmed that *Airn* was over-expressed in the liver fibrosis model (Fig. 3A). Over-expression of *Airn* greatly alleviated CCl_4_-induced liver fibrosis as demonstrated by macroscopic examination, H&E staining, sirius red staining, serum ALT, AST level, and liver hydroxyproline content (Fig. 3B and Additional file 1: Table S4). Moreover, *Airn* over-expression markedly suppressed upregulation of CCl_4_-induced α-SMA and COL1α1, whereas obviously promoted compensatory upregulation of PCNA by IHC (Fig. 3B). In addition, qRT-PCR and western blot analysis showed that over-expression of *Airn* suppressed upregulation of CCl_4_-induced α-SMA, COL1α1, MMP2, and TIMP1 (Fig. 3C, D). On the other hand, SEM analysis indicated that the number of fenestrae was significantly decreased in CCl_4_-induced mice, while *Airn* over-expression rescued the reduction of fenestrae (Fig. 3E). Furthermore, *Airn* over-expression ameliorated CCl_4_-induced LSEC capillarization assessed by IHC and qRT-PCR for CD31 and LAMININ (Fig. 3C, E), demonstrating that *Airn* over-expression suppressed CCl_4_-induced LSEC capillarization. Taken together, these results confirmed that over-expression of *Airn* alleviated CCl_4_-induced liver fibrosis in vivo and was associated with LSEC capillarization*.*

### Airn maintained LSEC differentiation in vitro

**Fig. 4:**
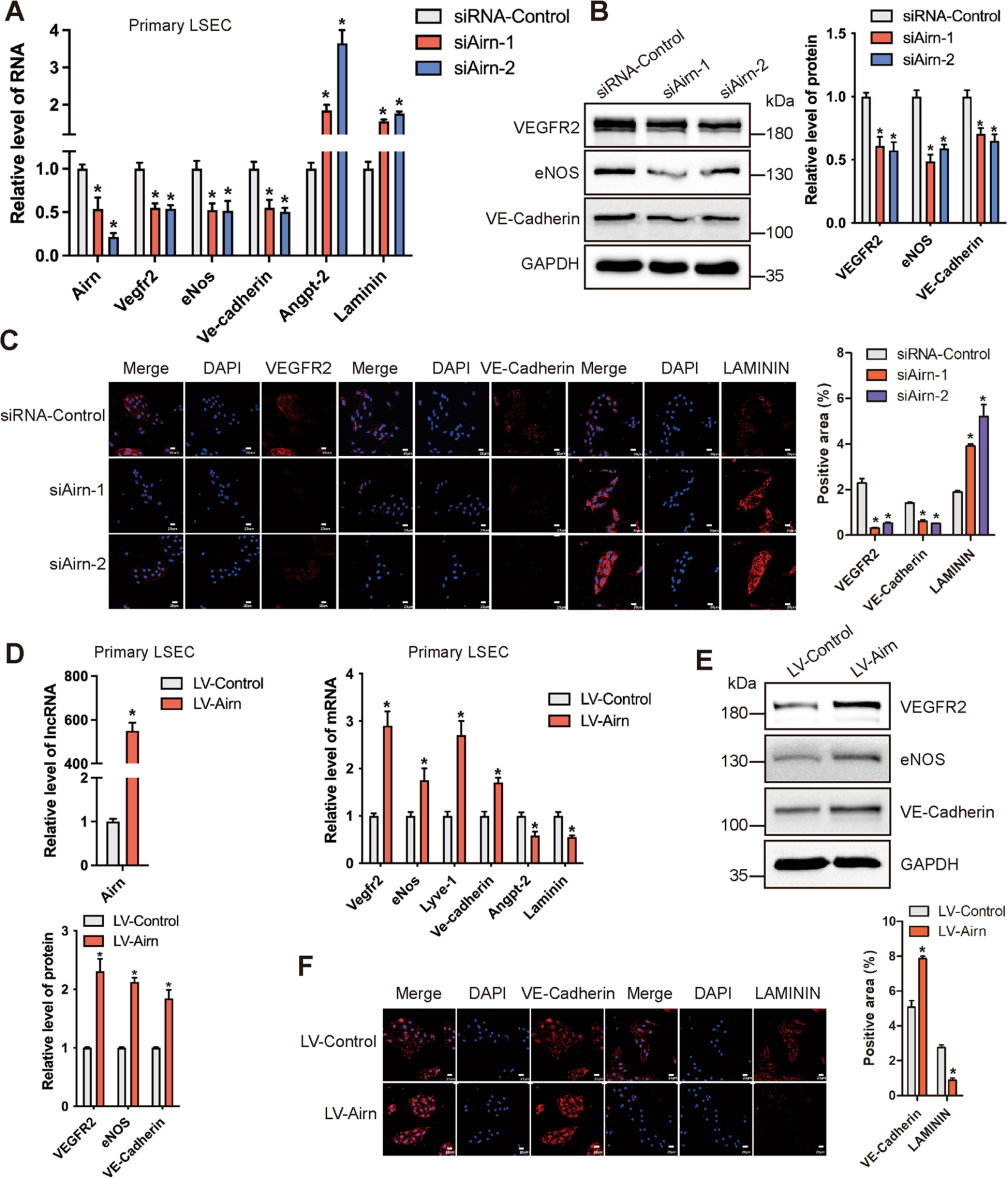
*Airn* maintained LSEC differentiation in vitro. **A** Primary LSECs were transfected with si*Airn*-1, si*Airn*-2, , 20μm. **D** Primary LSECs were infected with LV-*Airn* and LV-Control for 72 h. The RNA level of *Airn*, *Vefgr2*, *eNos*, *Lyve-1*, *VE-cadherin*, *Angpt-2*, and *Laminin* was detected by qRT-PCR. **E** The protein level of VEGFR2 and eNOS was determined by western blot and quantitatively compared with GAPDH as a reference control. **F** The expression of VE-Cadherin and LAMININ was determined by confocal microscopy and quantitatively compared. DAPI-stained nuclei blue; scale bar, 20 μm. The data are expressed as the mean ± SD for at least triplicate experiments, *p<0.05 stands for vs siRNA-Control or LV-Control

The in vivo data showed that *Airn* alleviated CCl_4_-induced LSEC capillarization. Therefore, to investigate whether *Airn* was involved in LSEC capillarization and differentiation in vitro, we first synthesized three specific siRNAs against *Airn* (si*Airn*-1, si*Airn*-2, and si*Airn*-3). Among them, si*Airn*-1 and si*Airn-2* showed an efficient knockdown effect and were applied in subsequent experiments. RNA-seq analysis showed that knockdown of *Airn* decreased LSEC differentiation-associated genes including *Flt4 (Vegfr3)*, *Lyve-1*, and *Stab1*, but increased LSEC capillarization-associated genes including *Gabre*, *Lama1*, and *Lama2* [23] (Additional file 1: Fig. S6A). qRT-PCR analysis further verified that knockdown of *Airn* significantly reduced *Vegfr2*, *eNos*, *Lyve-1*, and *VE-cadherin* expression, whereas markedly enhanced *Laminin* and *Angpt2* expression (Fig. 4A). Moreover, the protein level of VEGFR2, eNOS, and VE-Cadherin was markedly decreased in *Airn*-silenced LSECs assessed by western blot and confocal microscopy (Fig. 4B, C), indicating that *Airn* silencing promoted LSEC capillarization. On the other hand, over-expression of *Airn* remarkably enhanced the expression of *Vegfr2*, *eNos*, *Lyve-1*, and *VE-cadherin*, while significantly suppressed the expression of *Laminin* and *Angpt2* in primary LSEC (Fig. 4D). Consistently, the protein level of VEGFR2, eNOS, and VE-Cadherin was notably increased in *Airn* over-expressing LSEC (Fig. 4E, F). Additionally, the expression of *Vegfr2* and *eNos* in the primary LSECs isolated from AAV8-*Airn* mice exhibited remarkable enhancement, but the expression of *Laminin* demonstrated a profound reduction, in comparison with the AAV8-*GFP* mice (Additional file 1: Fig. S6B). Taken together, our results demonstrated that *Airn* maintained LSEC differentiation in vitro.

### Airn inhibited HSC activation indirectly through maintaining LSEC differentiation

**Fig. 5:**
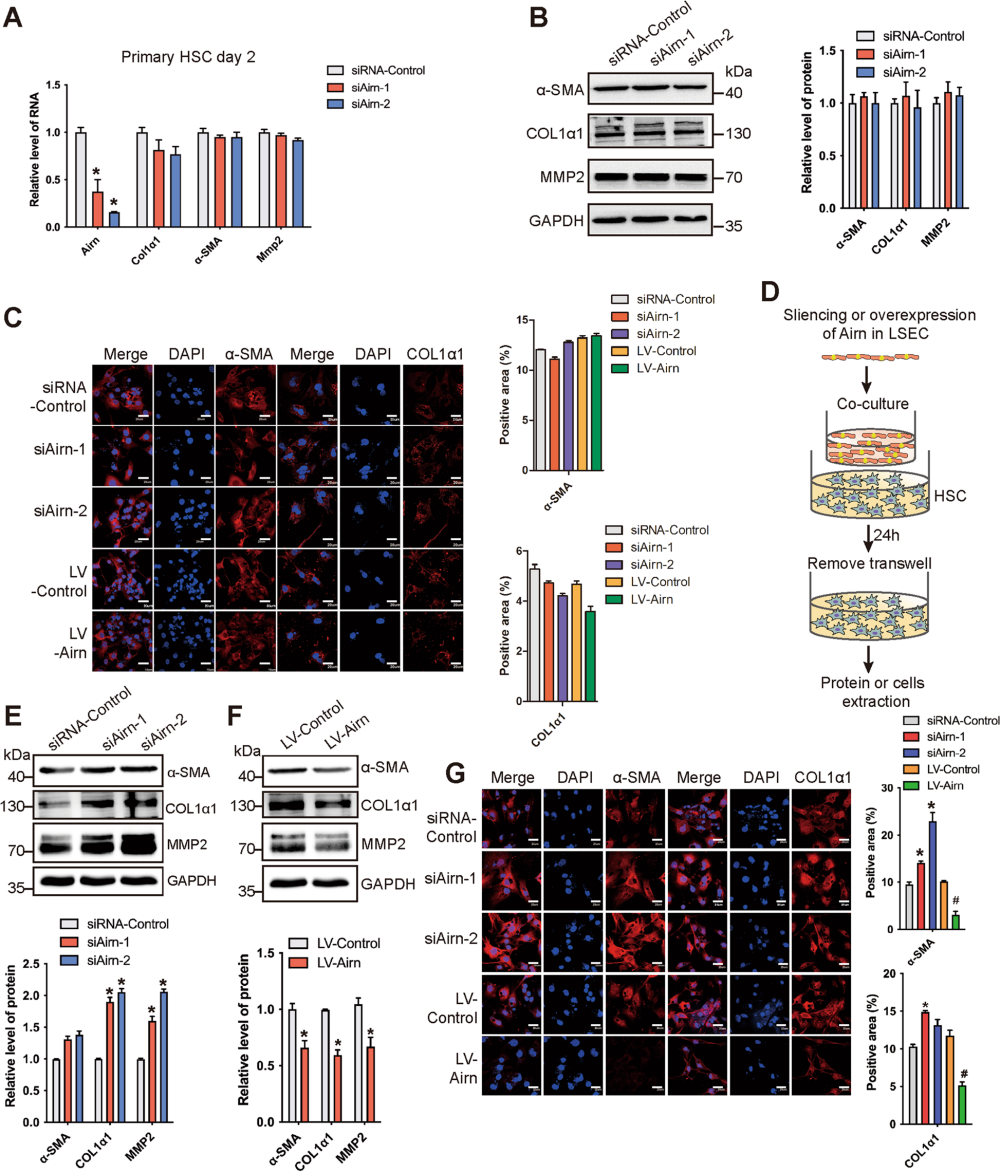
*Airn* inhibited HSC activation indirectly through maintaining LSEC differentiation. **A** Primary HSCs at day 2 were transfected with si*Airn*-1, si*Airn*-2, or siRNA-Control for 48 h. The RNA level of *Airn*, *α-SMA*, *Col1α1*, and *Mmp2* was detected by qRT-PCR. **B** The protein level of α-SMA, COL1α1, and MMP2 was determined by western blot and quantitatively compared with GAPDH as a reference control. **C** The expression of α-SMA and COL1α1 was determined by confocal microscopy and quantitatively compared. DAPI-stained nuclei blue; scale bar, 20 μm. **D** Schematic diagram illustrating the design of the co-culture experiments. The primary HSCs derived from health mice were co-cultured with the *Airn*-silenced or -over-expressed of primary LSECs by transwell, or co-cultured with untreated LSEC. **E, F** The expression of α-SMA, COL1α1, and MMP2 in co-cultured with the *Airn*-silenced or -over-expressed LSECs was determined by western blot and quantitatively compared with GAPDH as a reference control. **G** The expression of α-SMA and COL1α1 in co-cultured with the *Airn*-silenced or -over-expressed LSECs was determined by confocal microscopy and quantitatively compared. DAPI-stained nuclei blue; scale bar, 20 μm. The data are expressed as the mean ± SD for at least triplicate experiments, *p<0.05 stands for vs siRNA-Control or LV-Control

HSC activation have been commonly recognized as the principal cellular players promoting synthesis and deposition of ECM. The expression of marker genes including collagens I and III, α-SMA, MMPs, and TIMPs are significantly increased in the activated HSC compared with that of quiescent HSCs. Additionally, it has been reported that isolated HSCs remains quiescent when cultured for 3 days in vitro, became partly activated at day 7 and fully activated at day 14 [24]. Since the results in vivo showed that *Airn* alleviated CCl_4_-induced liver fibrosis, the effect of *Airn* was investigated in HSCs. However, the expression of COL1α1, α-SMA and MMP2 was unchanged in *Airn*-silenced or -over-expressed primary HSC at day 2 assessed by qRT-PCR, western blot, and confocal microscopy (Fig. 5A–C and Additional file 1: Fig. S7A-B). Similarly, these results were confirmed in primary HSCs at day 12 (Additional file 1: Fig. S7C-F) suggesting that *Airn* was not directly involved in the regulation of HSC activation. Therefore, we hypothesized that *Airn* indirectly acted on HSC by maintaining LSEC differentiation. Subsequently, *Airn*-silenced or -over-expressed primary LSEC was used to co-culture with primary HSC (Fig. 5D). The expression of the fibrotic genes including α-SMA and COL1α1 was significantly upregulated in HSCs co-culturing with *Airn*-silenced primary LSECs compared with that treated with the control LSECs (Fig. 5E, G), while the expression of these genes was repressed in HSC co-culturing with *Airn*-over-expressed primary LSEC assessed by western blot and confocal microscopy (Fig. 5F, G). Taken together, these results suggested that *Airn* inhibited HSC activation indirectly through maintaining LSEC differentiation.

### Airn promoted HC proliferation directly and indirectly by paracrine secretion of Wnt2a and HGF from LSECs

**Fig. 6:**
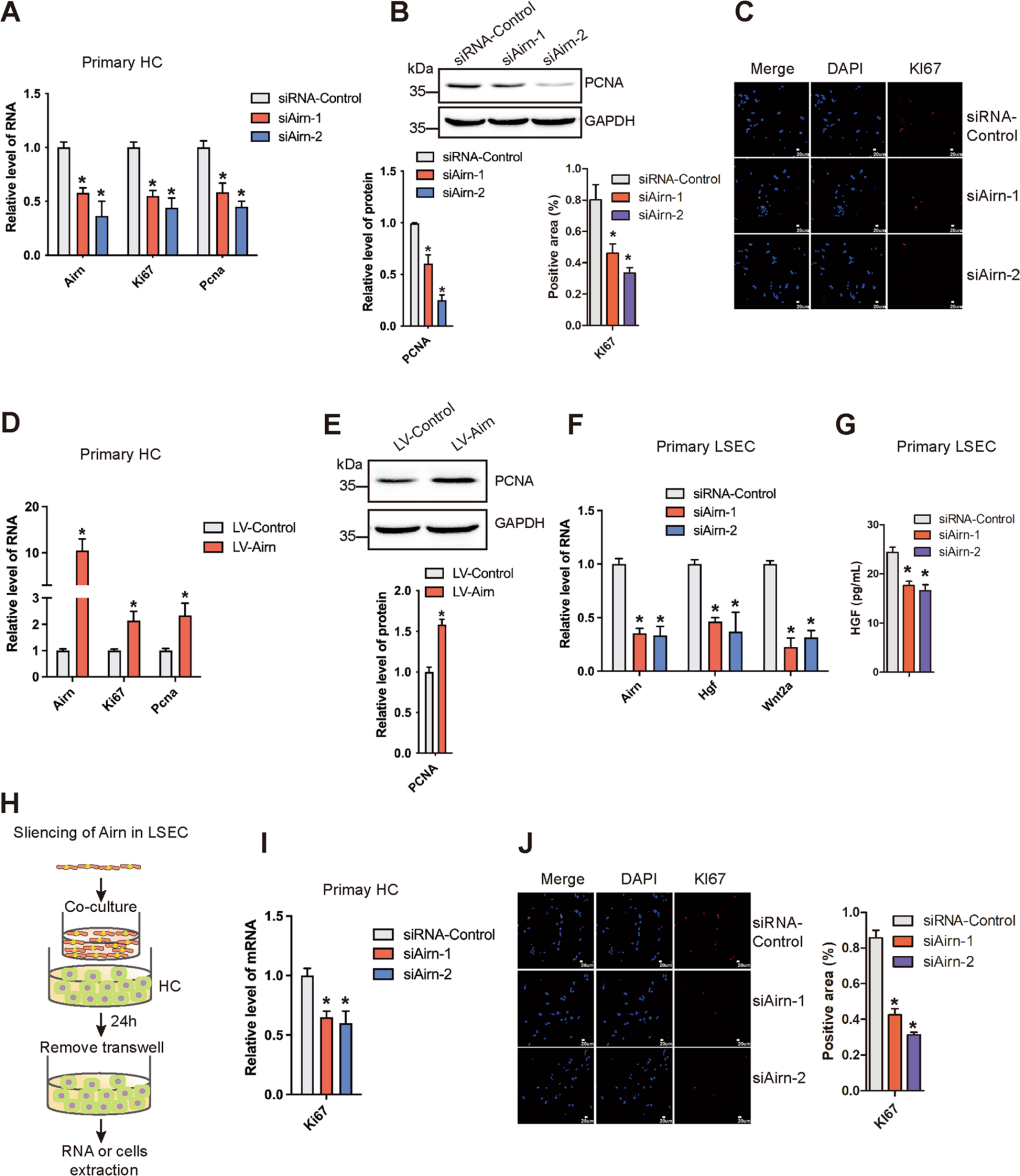
*Airn* promoted HC proliferation directly and indirectly by paracrine secretion of Wnt2a and HGF from LSEC. **A** Primary HCs were transfected with si*Airn*-1, si*Airn*-2, or siRNA-Control for 48 h. The RNA level of *Airn*, *Ki67*, and *Pcna* was detected by qRT-PCR. **B** The protein level of PCNA was determined by western blot and quantitatively compared with GAPDH as a reference control. **C** The expression of Ki67 was determined by confocal microscopy and quantitatively compared. DAPI-stained nuclei blue; scale bar, 20μm. **D** Primary HC were infected with LV-*Airn* and LV-Control for 72 h. The RNA level of *Airn*, *Ki67*, and *Pcna* was detected by qRT-PCR. **E** The protein level of PCNA was determined by western blot and quantitatively compared with GAPDH as a reference control. **F** Primary LSEC were transfected with si*Airn*-1, si*Airn*-2, or siRNA-Control for 48 h. The RNA level of *Wnt2a* and *HGF* was detected by qRT-PCR. **G** *Airn* was silenced by siRNA in primary LSEC and cultured in vitro for 48 h, the expression of HGF was detected in culture supernatants by ELISA. **H** Schematic diagram illustrating the design of the co-culture experiments. The primary HC derived from health mice were co-cultured with the Airn-silenced or -over-expressed of primary LSEC by transwell, or co-cultured with untreated LSEC. **I** The expression of *Ki67* was detected by qRT-PCR in co-cultured condition. **J** The expression of Ki67 was determined by confocal microscopy in co-cultured condition and quantitatively compared. DAPI-stained nuclei blue; scale bar, 20μm. The data are expressed as the mean ± SD for at least triplicate experiments, *p<0.05 stands for vs siRNA-Control or LV-Control

To investigate whether *Airn* was required for the proliferation of HC in vitro, we knocked down the expression of *Airn* by siRNA in primary HC and AML12 cells. qRT-PCR, western blot, and confocal microscopy analysis showed that the expression of Ki67 and PCNA was significantly decreased in *Airn*-silenced primary HCs or AML12 cells (Fig. 6A–C and Additional file 1: Fig. S8A-C). CCK8 assay showed that *Airn*-silenced notably inhibited cell proliferation (Additional file 1: Fig. S8D). Moreover, over-expression of *Airn* remarkably increased the expression of Ki67 and PCNA in primary HCs and AML12 cells (Fig. 6D,E and Additional file 1: Fig. S8E-G). CCK8 and EdU assay showed that over-expression of *Airn* significantly improved cell proliferation (Additional file 1: Fig. S8H, I). Additionally, the expression of the pro-proliferation genes exhibited a profound reduction in the primary HCs isolated from CCl_4_-induced *Airn*-KO mice, in comparison with that of the CCl_4_-induced WT mice (Additional file 1: Fig. S9A, B), indicating that *Airn* promoted HC proliferation directly. While it has been reported that LSECs promoted HC proliferation by paracrine hepatic cytokines including Wnt2a and HGF [7, 21]. Therefore, we investigated whether *Airn* promoted HC proliferation by LSECs paracrine signal. The results showed that *Airn* silencing inhibited the mRNA of expression of *Hgf* and *Wnt2a* in primary LSEC (Fig. 6F). To further detect whether *Airn* regulated LSEC paracrine secretion, *Airn* was silenced by siRNA in primary LSECs and cultured in vitro for 48 h, and the level of HGF was detected in culture supernatants by ELISA. The data showed that *Airn* silencing downregulated the secretion of HGF in LSEC (Fig. 6G). Next, *Airn*-silenced primary LSEC were used to co-culture with primary HC (Fig. 6H). Compared with the control LSEC-treated HC, the expression of Ki67 was significantly reduced in HC when co-cultured with *Airn*-silenced primary LSECs by qRT-PCR and confocal microscopy (Fig. 6I, J). Taken together, *Airn* not only directly promoted the proliferation of HC, but also promoted the proliferation of HCs through LSEC paracrine secretion of Wnt2a and HGF.

### Airn maintained LSEC differentiation through the KLF2-eNOS-sGC pathway

**Fig. 7:**
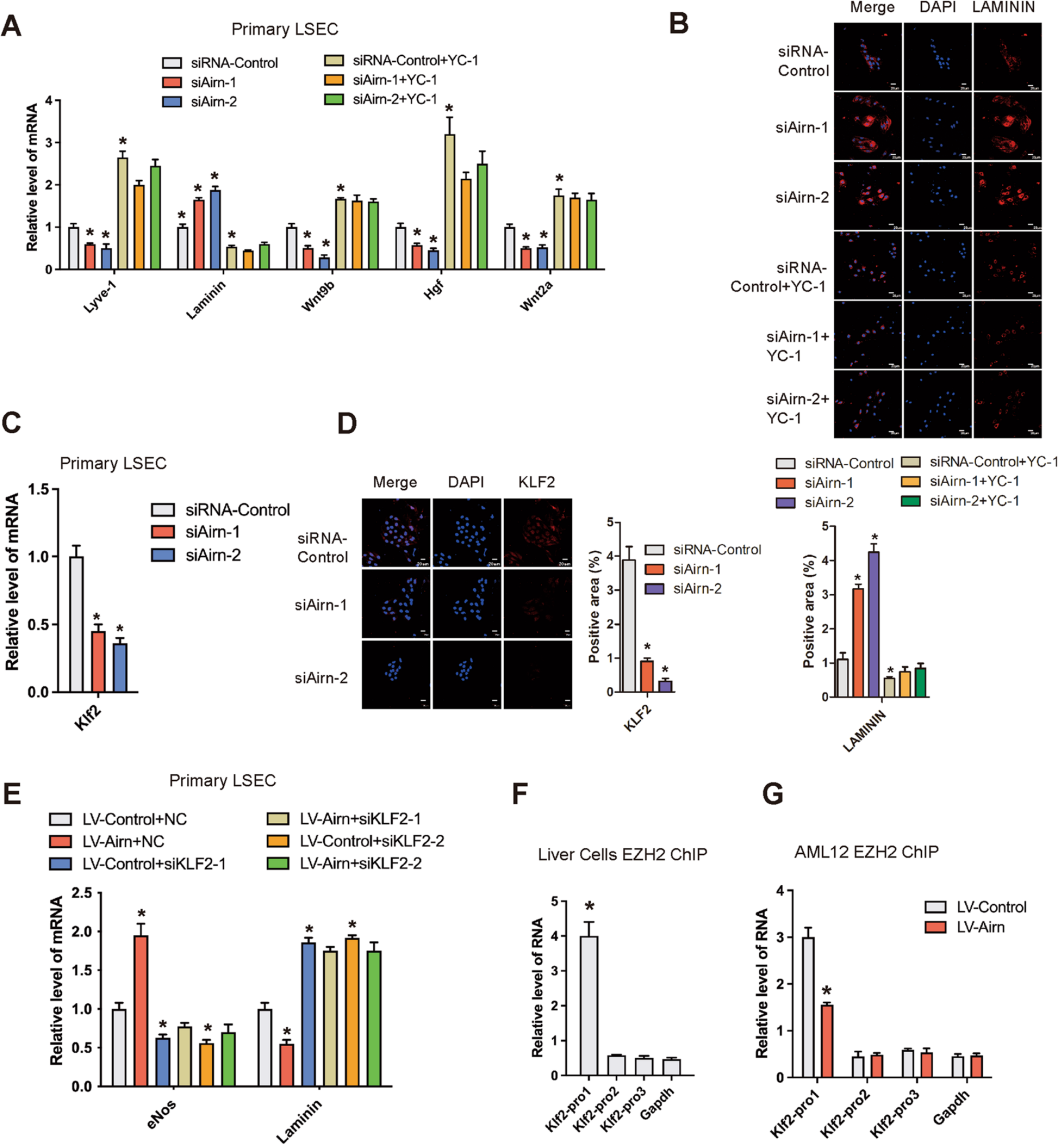
*Airn* maintained LSEC differentiation through KLF2-eNOS-sGC pathway. Primary LSEC were transfected with si*Airn* or siRNA-Control for 48 h and further treated with 30 μM YC-1 for additional 24 h. **A** The expression of *Lyve-1*, *Laminin*, *Wnt9b*, *HGF*, and *Wnt2a* was detected by qRT-PCR. **B** The expression of LAMININ was detected by confocal microscopy and quantitatively compared. DAPI-stained nuclei blue; scale bar, 20 μm. **C–E** Primary LSEC were transfected with si*Airn* or siRNA-Control for 48 h, the expression of KLF2 was detected by qRT-PCR (**C**), confocal microscopy (**D**) and quantitatively compared. DAPI-stained nuclei blue; scale bar, 20 μm. **E** Primary LSEC were transfected with LV-*Airn* or LV-Control for 48 h and further treated with siKLF2 for additional 48 h. The expression of *eNos* and *Laminin* was detected by qRT-PCR. All data are presented as means ± SD for at least triplicate experiments. **F** ChIP analyses were performed on indicated gene promoter regions using anti-EZH2 antibody in the single-cell suspensions of mouse liver. **G** AML12 cells were infected with LV-*Airn* or LV-Control, and ChIP analyses were performed on indicated genes promoter regions using anti-EZH2 antibody. *p < 0.05 stands for vs siRNA-Control or LV-Control + NC

We next explore the mechanism of *Airn* in maintaining LSEC differentiation. It has been reported that the LSEC phenotype maintained, at least partly, through eNOS-sGC signaling [21] and we have revealed that *Airn* promoted the expression of eNOS and regulated the expression of eNOS-sGC downstream target genes (Fig. 4A–E). Therefore, LSEC were treated with sGC agonist YC-1. qRT-PCR and confocal microscopy analysis confirmed that *Airn* silencing decreased the expression of LYVE-1 and increased the expression of LAMININ, which was abrogated by YC-1 (Fig. 7A, B). Moreover, YC-1 rescued *Airn* silencing-induced downregulation of *Wnt2a*, *Hgf*, and *Wnt9b*, indicating that the knockdown of *Airn* in LSEC suppressed HC proliferation via the eNOS-sGC pathway (Fig. 7A). Furthermore, primary LSEC was isolated from WT and *Airn*-KO mice, subsequently treated with YC-1. The expression of Lyve-1 was downregulated, while Laminin was upregulated in the primary LSEC isolated from *Airn*-KO mice compared with WT (Additional file 1: Fig. S10A, B). However, augmentation of *Laminin* induced by knockout of *Airn* was abolished by YC-1 (Additional file 1: Fig. S10A, B). In addition, the expression of KLF2, which has been reported to have positively regulated the transcription of eNOS [25, 26], was decreased in *Airn*-silenced primary LSEC (Fig. 7C, D and Additional file 1: Fig. S10C) and increased in *Airn*-over-expressed primary LSECs assessed by qRT-PCR, western blot, and confocal microscopy (Additional file 1: Fig. S10D-F). Thus, to investigate whether *Airn* regulated the eNOS-sGC pathway via KLF2, specific siRNA targeting KLF2 was transfected in *Airn*-over-expressed primary LSEC and the results showed that KLF2 silencing abrogated *Airn* over-expression-induced upregulation of *eNos* and downregulation of *Laminin* assessed by qRT-PCR (Fig. 7E). Taken together, our data demonstrated that *Airn* maintained LSEC differentiation through the KLF2-eNOS-sGC pathway, thereby inhibiting HSC activation and HC proliferation.

Generally, lncRNAs regulate their target genes by interacting with RNA binding proteins or acting as endogenous competing RNAs for specific microRNAs. To understand the molecular mechanism underlying the effects of *Airn* on liver fibrosis and LSEC capillarization, we first investigated the cell distribution of *Airn* using FISH and qRT-PCR. The results showed that *Airn* was mainly located in the nucleus of primary LSECs and HCs (Additional file 1: Fig. S11A-C). Moreover, our data demonstrated that *Airn* promoted the expression of KLF2 at both protein and RNA level in primary LSEC. Thus, a bioinformatics tool was used to screen for *Airn*-interacting proteins. The data showed that the 58 proteins including EZH2, which has been reported to bind to the promoter regions of KLF2 [27, 28], possibly interact with both human and mouse *Airn* (Additional file 1: Fig. S11D-G). To investigate this interaction, RIP assay was performed and the results showed a significant enrichment of *Airn* with the EZH2 antibody (Additional file 1: Fig. S12A, B). In addition, to identify the exact region of the *Airn* responsible for EZH2 interaction, we constructed the full-length *Airn*, a series of truncated *Airn* and the indicated antisense probe (Additional file 1: Fig. S12C). The results of RNA pull-down indicated that nucleotides 532 to 869 of *Airn* could bind EZH2, consistent with the prediction (Additional file 1: Fig. S12D). Moreover, ChIP analysis demonstrated that EZH2 could bind to promoter regions of KLF2 and over-expression of *Airn* reduced their binding ability (Fig. 7F, G). Taken together, these data demonstrated that *Airn* interacts with EZH2 and may block the binding site of EZH2 to KLF2, thus releasing the inhibition of KLF2 and LSEC differentiation related genes.

## Discussion

Liver fibrosis represents the consequence of a sustained healing response originating from chronic injury. It is characterized by excessive accumulation of extracellular matrix components and can eventually lead to cirrhosis [2, 29]. Angiogenesis with an abnormal angioarchitecture is a hallmark related to liver fibrogenesis, which implicates a potential target for therapeutic interventions [30]. However, the occurrence of angiogenesis in liver fibrosis remains controversial. For instance, Taura et al. demonstrated that CD31, a marker for endothelial cells, was increased as fibrosis developed [31]. Moreover, Lao et al. investigated the expression of VEGF was continuously increased during sustained damage for CCl_4_, suggesting that liver fibrosis is accompanied by increased vascular density [32]. However, Liu et al. suggested that angiogenesis increased sharply at the early stage of liver fibrosis, while gradually diminished along with the formation of insoluble scars in late-stage fibrotic livers [30]. In this study, we also found that the expression of angiogenesis markers CD34 and CD31 in human and mouse fibrotic livers were significantly increased in mild fibrosis and drastically reduced in advanced fibrosis (Additional file 1: Fig. S13A, B). Moreover, our study revealed that the role of *Airn* in LSEC is to repress capillarization and might be stimulated to over-express to inhibit CD34 and CD31, maintaining the differentiation. This could explain the result that *Airn* expression levels was significantly upregulated in mild fibrotic liver samples but not in advanced fibrotic liver tissues. All these data supported our conclusion that *Airn* played an important role in the angiogenesis in liver fibrosis.

LSEC are the most numerous non-parenchymal cells in the liver; therefore, they are destined to play an irreplaceable role in various liver diseases. Capillarized LSEC have been shown to precede fibrosis and promote HSC activation and hepatocyte damage [8]. Furthermore, LSEC are important for acting as autocrine and paracrine source for signals of liver fibrosis [33]. It has been reported that LSEC secrete Wnt2 and HGF and promote hepatocyte proliferation and liver regeneration [7]. Moreover, differentiated LSEC can maintain HSC quiescence by producing NO [6], HB-EGF [34], and SK1 [35], while capillarized LSEC lose their capacity to inactivate HSC by secreting EIIA-fibronectin [36], PDGF, TGF-β [37], TNF-α, and IL1 [32], thus promoting fibrogenesis. Therefore, targeting LSEC has great therapeutic prospect for liver fibrosis. In the current study, we confirmed that *Airn* deficiency aggravated CCl_4_- and BDL-induced LSEC capillarization, while over-expression of *Airn* alleviated CCl_4_-induced LSEC capillarization in vivo*.* Moreover, over-expression of *Airn* remarkably enhanced the expression of VEGFR2, eNOS, and LYVE-1; meanwhile, it significantly suppressed the expression of LAMININ and ANGPT2 in primary LSEC in vitro. Further study indicated that *Airn* inhibits HSC activation indirectly by regulating LSEC differentiation and promoting HC proliferation directly and indirectly by the increased paracrine secretion of Wnt2a and HGF from LSEC, providing the proof that *Airn* plays a vital role in the orchestration of LSEC/HSC/HC interaction during liver fibrosis and *Airn* repressed liver fibrosis mainly via inhibiting LSEC capillarization. In addition, VE-cadherin (CD144) is an endothelial specific adhesion molecule locating at endothelial cell junctions and plays a role for paracellular permeability and maintenance of cell polarity [38]. Cyrill et al. revealed that capillarization was characterized by ectopic basement membrane deposition, formation of a continuous EC layer, and increased expression of VE-cadherin [39]. However, Alessio et al. revealed VE-cadherin expression was reduced by inhibitors of NOS [40]. It has been also reported that VE-cadherin could inhibit proliferation in part by altering cytoskeletal structure and decreasing cell spreading [41]. Interestingly, in our study, the results demonstrated that knockdown of *Airn* significantly reduced VE-cadherin expression, and over-expression of *Airn* remarkably enhanced the expression of VE-cadherin. However, the mechanism by which *Airn* regulate VE-cadherin still needs further investigation.

Currently, a mechanism discovered to date is that lncRNA could serve as molecular scaffold and recruit proteins or RNAs to target genes, thereby exerting biological functions. It has been reported that about 20% of lncRNAs, such as HEIH, XIST, KCNQ1OT1, and HOTAIR, expressed in various cell types are bound by PRC2, suggesting that these lncRNAs may have a general role in recruiting PRC2 to their target genes [42]. Emerging evidence suggests lncRNAs could bind to PRC2 and directly regulate the expression of specific genes, for instance, LINC00673 repressed LATS2 expression by recruiting PRC2 to the promoter, subsequently promoting gastric cancer development and progression [27], and lncRNA GHET1 could epigenetically repress transcription of KLF2 by recruiting PRC2 to KLF2 promoter in hepatocellular carcinoma cells [28]. In addition, lncRNAs can also act as endogenous competing RNAs to regulate gene expression, for example, SCARNA10 interacted with PRC2 and blocked PRC2-mediated repression of pro-fibrogenic genes expression [19]. Lethe interacts with NF-κB subunit RelA to inhibit RelA DNA binding and target gene activation [43]. *Airn* may use the same mechanism as SCARNA10 and Lethe do to promote the expression of KLF2, functioning as a decoy lncRNA. In this article, the results showed that *Airn* physically interacts with EZH2, thus promoting the expression of LSEC differentiation and capillarization related genes. The mechanism could be that *Airn* bound to PRC2 competitively, thus blocking the PRC2 binding sites with the target genes and releasing PRC2 inhibition of KLF2 and LSEC differentiation related genes.

## Conclusions

In conclusion, we identified that *Airn* was increased in human and mice fibrotic livers and revealed that *Airn* deficiency aggravated CCl_4_- and BDL-induced liver fibrosis in vivo. Over-expression of *Airn* suppressed CCl_4_-induced liver fibrosis in vivo. Additionally, *Airn* maintained LSEC differentiation in vivo and in vitro. Furthermore, *Airn* inhibited HSC activation indirectly and promoted HC proliferation by paracrine secretion of Wnt2a and HGF from LSEC. Mechanistically, the results demonstrated that *Airn* interacted with EZH2 to maintain LSEC differentiation through KLF2-eNOS-sGC pathway, thereby promoting HSC quiescence and HC proliferation (Additional file 1: Fig. S14). Our work identified that *Airn* was beneficial to liver fibrosis by maintaining LSEC differentiation and might be a serum biomarker for liver fibrogenesis.

## Supplementary Information

**Additional file 1: Fig. S1,** related to Fig. 1. **Fig. S2**, related to Fig. 1. **Fig. S3**, related to Fig. 2. The construction and identification of *Airn* knockout mice. **Fig. S4**, related to Fig. 2. Histological examination of major internal organs from *Airn*-KO and WT mice. **Fig. S5**, *Airn* deficiency aggravated BDL-induced liver fibrosis and LSEC capillarization *in vivo*. **Fig. S6**, related to Fig. 4. **Fig. S7**, related to Fig. 5. *Airn* was not directly involved in the regulation of HSC activation. **Fig. S8**, related to Fig. 6. *Airn* promoted AML12 cells proliferation directly. **Fig. S9**, related to Fig. 6. **Fig. S10**, related to Fig. 7. **Fig. S11**, related to Fig. 7. **Fig. S12**, related to Fig. 7. *Airn* interacted with EZH2. **Fig. S13.** The correlation between *AIRN* level and angiogenesis or fibrosis. **Fig. S14.** Schematic diagram illustrates the role and mechanism of *Airn* in the differentiation of LSEC and liver fibrosis. **Table S1.** Baseline characteristics of patients with fibrotic liver serum. **Table S2.** Serum ALT, AST and liver hydroxyproline levels in CCl_4_-induced liver fibrosis model. **Table S3.** Serum ALT, AST and liver hydroxyproline levels in BDL-induced liver fibrosis model. **Table S4.** Serum ALT, AST and liver hydroxyproline levels in CCl_4_-induced liver fibrosis model.

**Additional file 2.**

## Data Availability

All data generated or analyzed during this study are included in this published article and its Supplementary information files. The transcriptome sequencing data have been deposited in GEO repository under accession number GSE174175. The dataset used and/or analyzed are available from the corresponding author on reasonable request.

## Abbreviations

Airn: Antisense Igf2r RNA
BDL: Bile duct ligation
CCl_4_: Carbon tetrachloride
eNOS: Nitric oxide synthase 3
HCC: Hepatocellular carcinoma
HCs: Hepatocytes
HSCs: Hepatic stellate cells
KLF2: Kruppel-like factor 2
LncRNAs: Long noncoding RNAs
LSECs: Liver sinusoidal endothelial cells
sGC: Soluble guanylate cyclase
