# Supplementary material for: LncRNA Airn maintains LSEC differentiation to alleviate liver fibrosis via the KLF2-eNOS-sGC pathway
Source: BMC Med. 2022 Sep 29;20:335. doi: 10.1186/s12916-022-02523-w (PMC9520944; doi:10.1186/s12916-022-02523-w)
Supplement: Supplementary file 1 — Additional file 1: Fig. S1, related to Fig. 1. Fig. S2, related to Fig. 1. Fig. S3, related to Fig. 2. The construction and identification of Airn knockout mice. Fig. S4, related to Fig. 2. Histological examination of major internal organs from Airn-KO and WT mice. Fig. S5, Airn deficiency aggravated BDL-induced liver fibrosis and LSEC capillarization in vivo. Fig. S6, related to Fig. 4. Fig. S7, related to Fig. 5. Airn was not directly involved in the regulation of HSC activation. Fig. S8, related to Fig. 6. Airn promoted AML12 cells proliferation directly. Fig. S9, related to Fig. 6. Fig. S10, related to Fig. 7. Fig. S11, related to Fig. 7. Fig. S12, related to Fig. 7. Airn interacted with EZH2. Fig. S13. The correlation between AIRN level and angiogenesis or fibrosis. Fig. S14. Schematic diagram illustrates the role and mechanism of Airn in the differentiation of LSEC and liver fibrosis. Table S1. Baseline characteristics of patients with fibrotic liver serum. Table S2. Serum ALT, AST and liver hydroxyproline levels in CCl4-induced liver fibrosis model. Table S3. Serum ALT, AST and liver hydroxyproline levels in BDL-induced liver fibrosis model. Table S4. Serum ALT, AST and liver hydroxyproline levels in CCl4-induced liver fibrosis model. [file 12916_2022_2523_MOESM1_ESM.docx]

**LncRNA *Airn* maintains LSEC differentiation to alleviate liver fibrosis via the KLF2-eNOS-sGC pathway**

Ting Chen^1†^, Zhemin Shi^1†^, Yanmian Zhao^1†^, Xiaoxiang Meng^1^, Sicong Zhao^1^, Lina Zheng^1^, Xiaohui Han^1^, Zhimei Hu^1^, Qingbin Yao^1^, Huajiang Lin^2^, Xiaoxiao Du^1^, Kun Zhang^1^*, Tao Han^2^*, Wei Hong^1^*

^1^Department of Histology and Embryology, School of Basic Medical Sciences, Tianjin Medical University, Tianjin, China

^2^Department of Hepatology and Gastroenterology, Tianjin Union Medical Center, Tianjin Medical University, Tianjin Union Medical Center affiliated to Nankai University, Tianjin, China

^†^ Ting Chen, Zhemin Shi and Yanmian Zhao contributed equally to this work.

Kun Zhang, Tao Han and Wei Hong are the lead contacts.

*Correspondence: [zhangkun@tmu.edu.cn](mailto:zhangkun@tmu.edu.cn); hantaomd@126.com; hongwei@tmu.edu.cn

**Supplementary Figures**


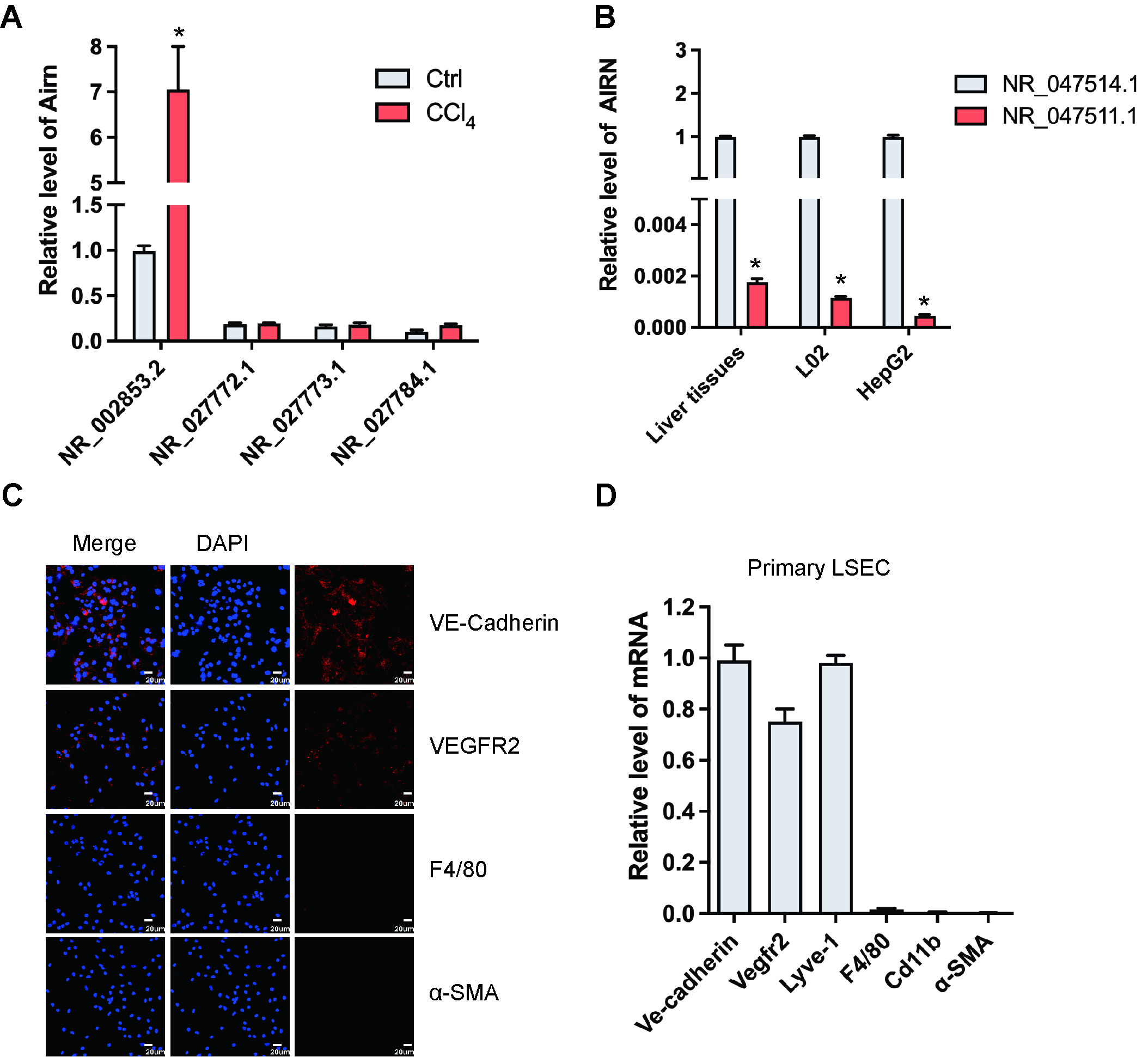


**Fig. S1, related to Fig. 1.** (A) The expression of various mouse *Airn* isoforms was detected in livers from control mice and CCl_4_-treated mice by qRT-PCR. (B) The expression of various human *AIRN* isoforms was detected in liver tissues, L02 and HepG2 cells by qRT-PCR. (C) Primary LSEC were cultured for 24 h in collagen coated plate and the purity was detected by confocal microscopy of VE-Cadherin and VEGFR2, α-SMA and F4/80. DAPI-stained nuclei blue; scale bar, 20μm. (D) The expression of *VE-cadherin*, *Vegfr2*, *Lyve1*, *F4/80*, *Cd11b* and *α-SMA* was analyzed by qRT-PCR. The data are expressed as the mean ± SD for at least triplicate experiments, **p*< 0.05.


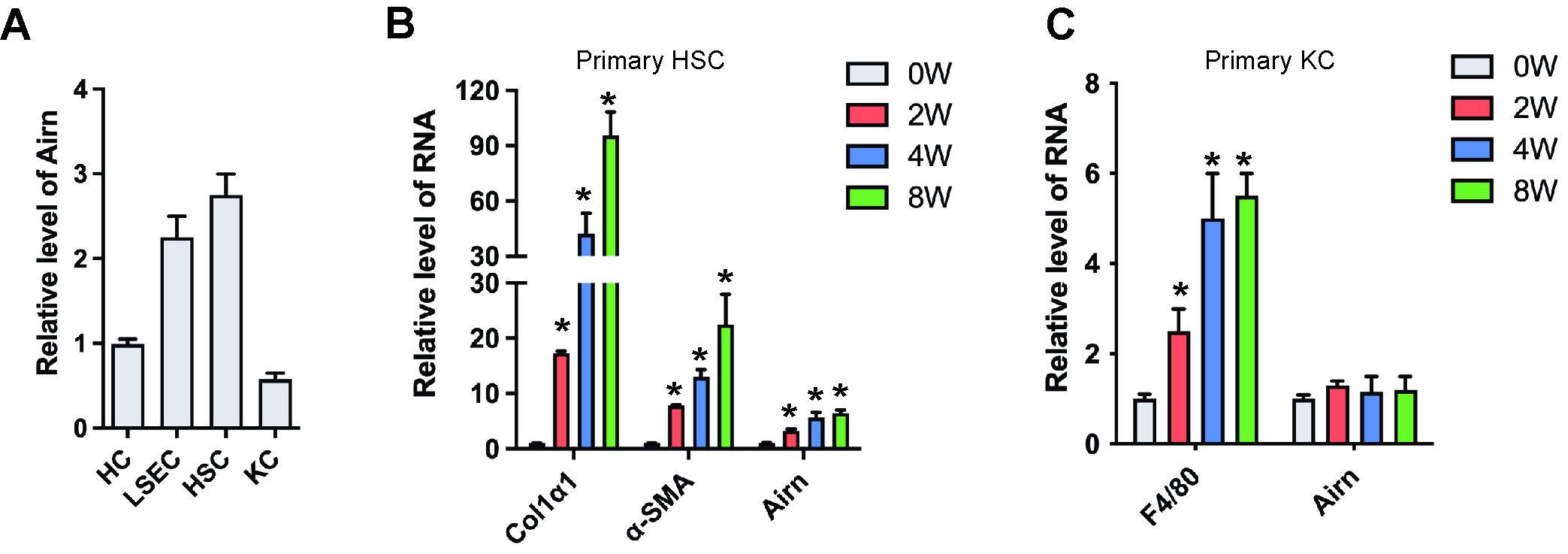


**Fig. S2, related to Fig. 1.** (A) HC, HSC, KC and LSEC were isolated from livers of mice, the expression of *Airn* was detected by qRT-PCR. (B) Primary HSC were isolated from livers of mice treated with CCl_4_ or oil for 0, 2, 4, 8 weeks. The expression of *α-SMA, Col1α1* and *Airn* was detected by qRT-PCR. (C) Primary KC were isolated from livers of mice treated with CCl_4_ or oil for 0, 2, 4 and 8 weeks. The expression of *F4/80* and *Airn* was detected by qRT-PCR. The data are expressed as the mean ± SD for at least triplicate experiments, **p*< 0.05.

**
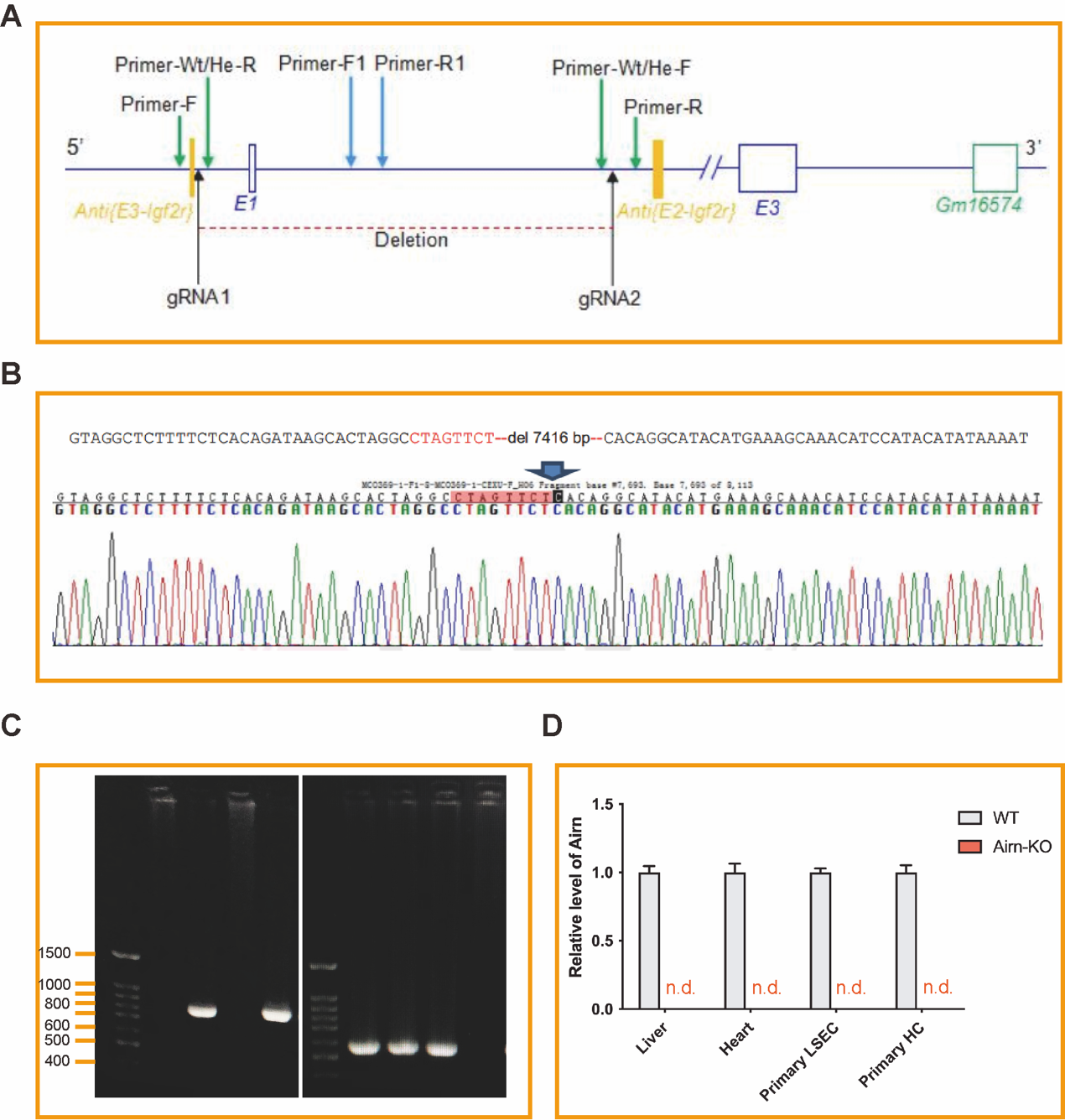
**

**Fig. S3, related to Fig. 2.** **The construction and identification of *Airn* knockout mice.** (A) The targeting strategy of *Airn* knockout (*Airn*-KO) mice via CRISPR/Cas9 system. (B, C) The positive animals were identified by sequencing (B) and PCR screening (C), the fourth sample, the product of 800 bp, was identified as homozygous. (D) The expression of *Airn* was detected in liver, heart, primary LSEC and primary HC isolated from WT and *Airn*-KO mice. The data are expressed as the mean ± SD for at least triplicate experiments.

**
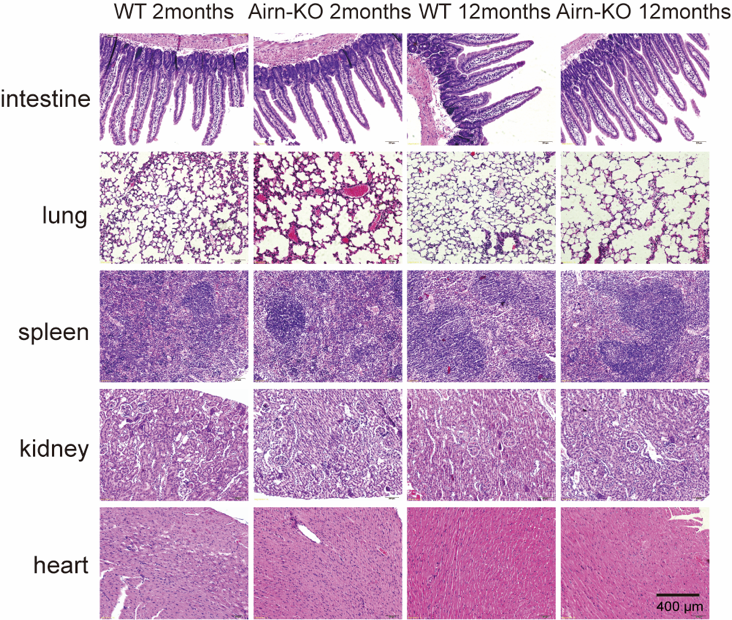
**

**Fig. S4, related to Fig. 2.** **Histological examination of major internal organs from *Airn*-KO and WT mice.** H&E staining was used to detect morphological abnormalities of different organs, including intestine, lung, spleen, kidney and heart from *Airn*-KO and WT mice at 2 or 12 months of age; scale bar, 400 μm for 10×.

**
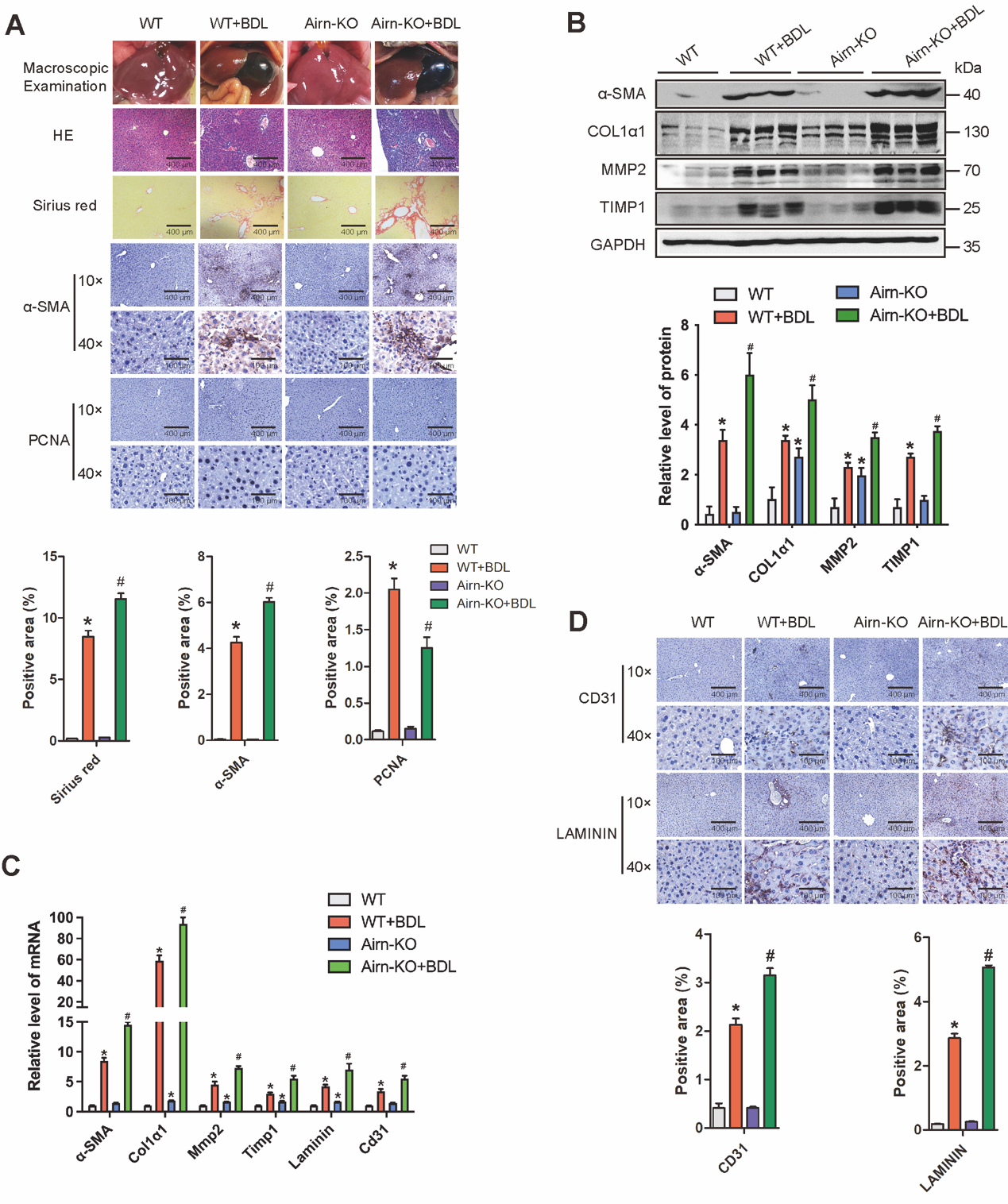
**

**Fig. S5,** ***Airn* deficiency aggravated BDL**-**induced liver fibrosis and LSEC capillarization *in vivo*.** C57BL/6 mice were divided into four groups: WT (n = 20), WT + BDL (n = 20), *Airn*-KO (n = 20) and *Airn*-KO + BDL (n = 20). (A) Liver fibrosis was evaluated by macroscopic examination, H&E staining, Sirius red staining and IHC for α-SMA and PCNA. Five images of each liver and five livers from different mice were quantified for each group; scale bar, 400 μm for 10× and 100 μm for 40×. (B) The protein level of α-SMA, COL1α1, MMP2 and TIMP1 were determined by western blot and quantitatively compared ﻿with GAPDH as a reference control.﻿ (C) The mRNA level of *α-SMA*, *Col1α1*, *Mmp2,* *Timp1*, *Laminin* and *Cd31* was determined by qRT-PCR. (D) The level of CD31 and LAMININ was detected by IHC staining ﻿and quantitatively compared; scale bar, 400 μm for 10× and 100 μm for 40×. **p*<0.05 stands for WT + BDL or *Airn*-KO vs WT. #*p*<0.05 stands for *Airn*-KO + BDL vs WT + BDL.


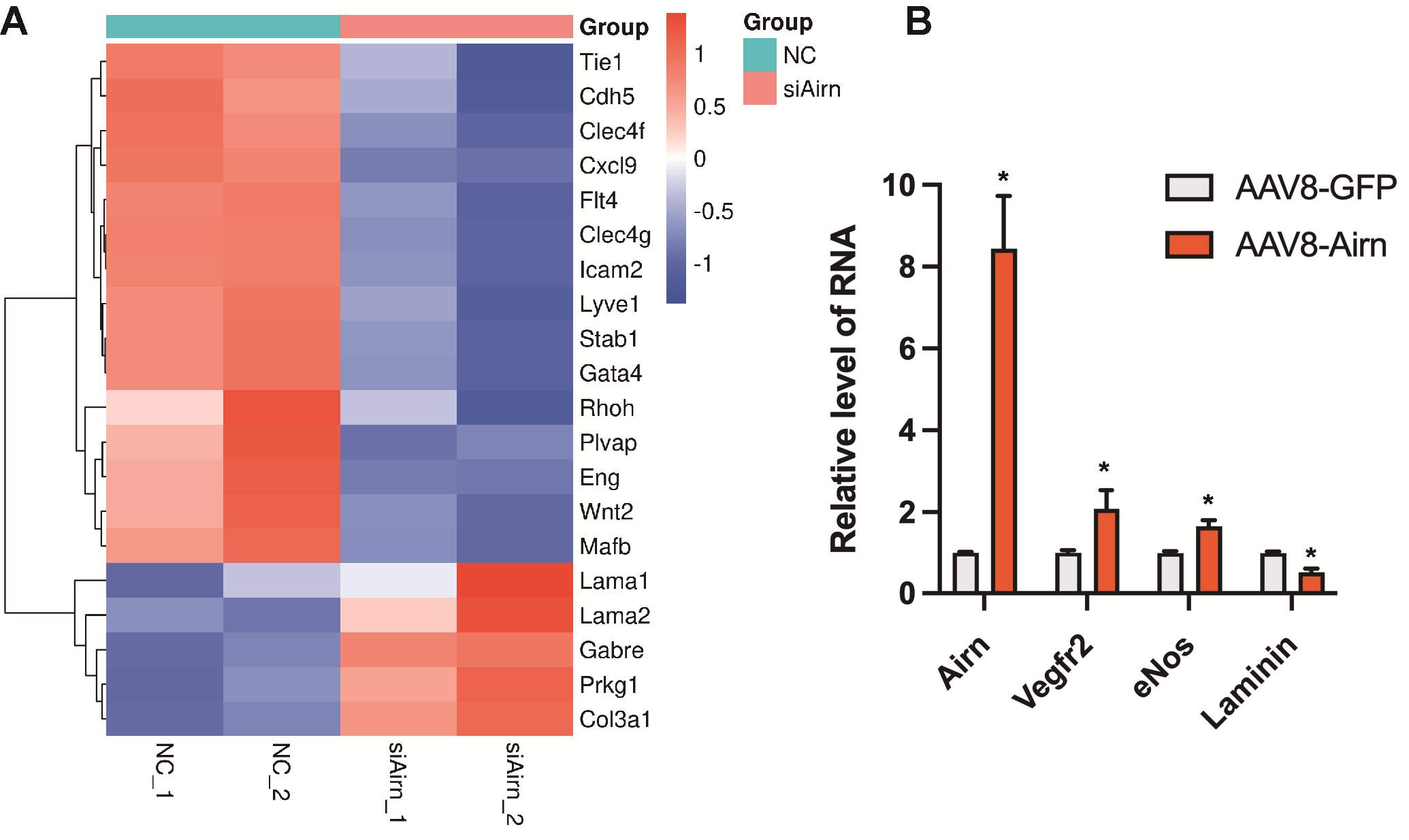


**Fig. S6, related to Fig. 4.** (A) Primary LSEC were infected with si*Airn*. Gene expression profiles were analyzed with RNA-seq, and the expression of LSEC-associated genes and continuous ECs-associated genes were displayed. The *p* value of each comparison was indicated with colors. (B) Balb/c mice (n=4) were injected by tail vein with 1x10^12^ pfu/mouse genome copies of AAV8-*GFP* or AAV8-*Airn*. After 10 days, LSEC were isolated and the RNA level of *Airn*, *Vefgr2*, *eNos* and *Laminin* was detected by qRT-PCR. The data are expressed as the mean ± SD for at least triplicate experiments, **p*<0.05 stands for vs AAV8-*GFP*.


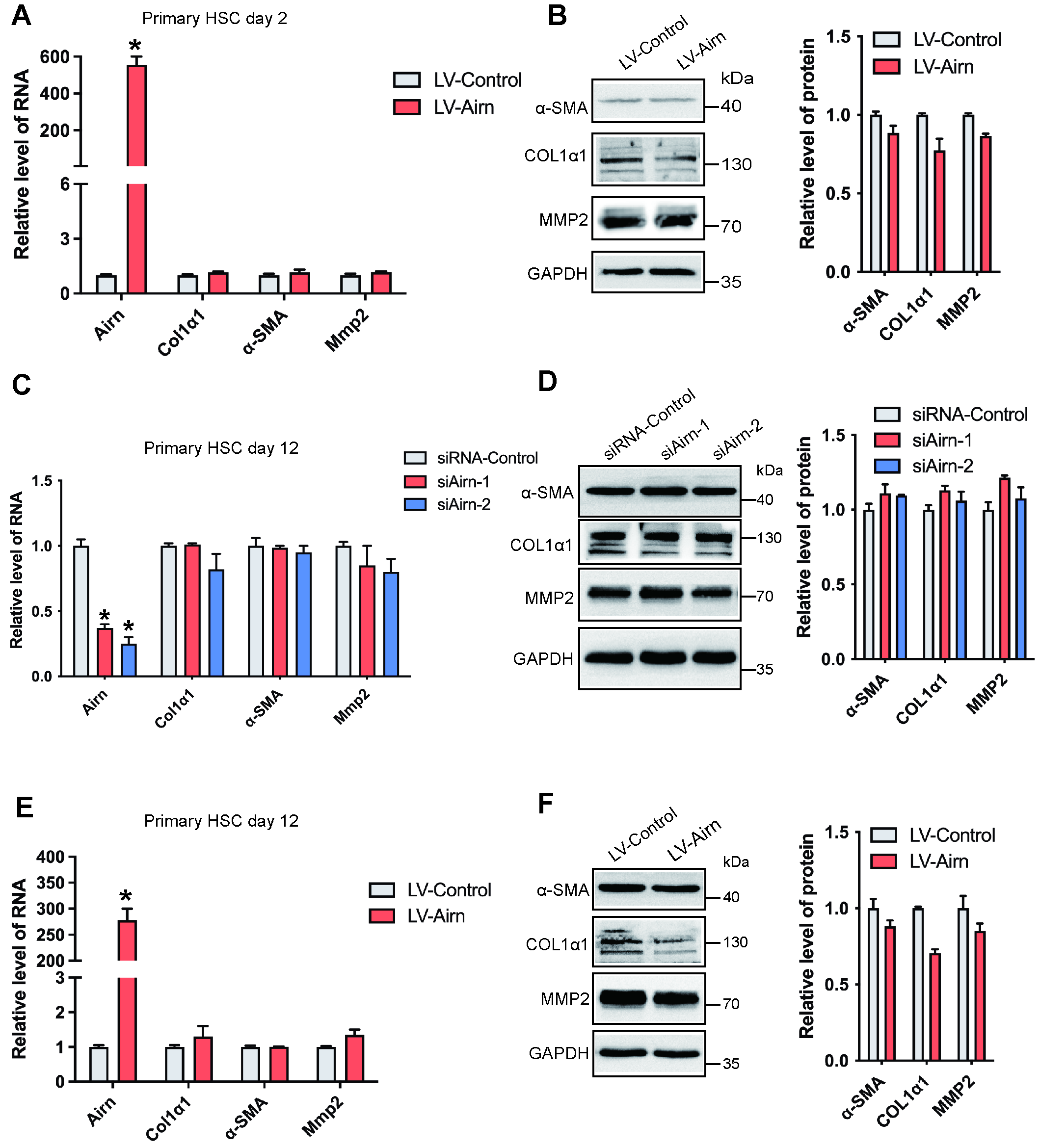


**Fig. S7, related to Fig. 5.** ***Airn* was not directly involved in the regulation of HSC activation.** (A) Primary HSC at day 2 were infected with LV-Airn and LV-Control for 72 h. The RNA level of *Airn*, *α-SMA*, *Col1α1* and *Mm*p2 was detected by qRT-PCR. (B) The protein level of α-SMA, COL1α1 and MMP2 was determined by western blot and quantitatively compared ﻿with GAPDH as a reference control. (C) Primary HSC at day 12 were transfected with si*Airn*-1, si*Airn*-2 or siRNA-Control for 48 h. The RNA level of *Airn*, *α-SMA*, *Col1α1* and *Mmp2* was detected by qRT-PCR. (D) The protein level of α-SMA, COL1α1 and MMP2 was determined by western blot and quantitatively compared, ﻿with GAPDH as a reference control.﻿ (E) Primary HSC at day 12 were infected with LV-*Airn* and LV-Control for 72 h. The RNA level of *Airn*, *α-SMA*, *Col1α1* and *Mmp2* was detected by qRT-PCR. (F) The protein level of α-SMA, COL1α1 and MMP2 was determined by western blot and quantitatively compared, ﻿with GAPDH as a reference control.﻿ The data are expressed as the mean ± SD for at least triplicate experiments. **p*<0.05 stands for vs siRNA-Control or LV-Control.

**
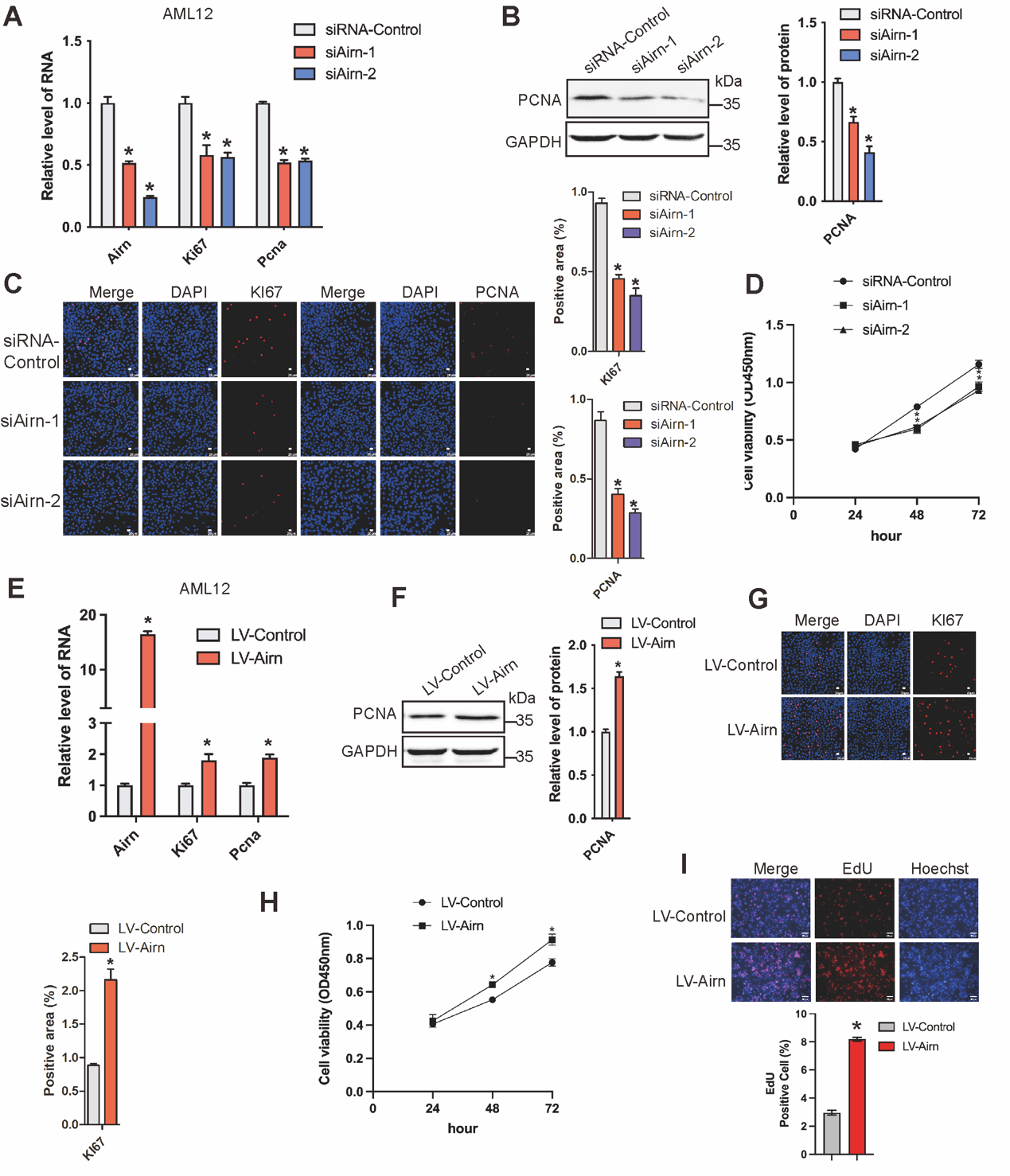
Fig. S8, related to Fig. 6.** ***Airn* promoted AML12 cells proliferation directly.** (A-D) AML12 cells were transfected with si*Airn*-1, si*Airn*-2 or siRNA-Control for 48 h. The RNA level of *Airn*, *Ki67* and *Pcna* was detected by qRT-PCR (A). The protein level of PCNA was determined by western blot and quantitatively compared ﻿with GAPDH as a reference control (B). The expression of Ki67 and PCNA was determined by confocal microscopy ﻿and quantitatively compared. DAPI-stained nuclei blue; scale bar, 20μm (C). CCK-8 assays were performed to evaluate the cellular growth curves in 24h, 48h, 72h (D). (E-H) AML12 cells were infected with LV-*Airn* and LV-Control for 72h. The RNA level of *Airn*, *Ki67* and *Pcna* was detected by qRT-PCR (E). The protein level of PCNA was detected by western blot and quantitatively compared ﻿with GAPDH as a reference control (F). The expression of Ki67 was determined by confocal microscopy ﻿and quantitatively compared (G), DAPI-stained nuclei blue; scale bar, 20μm. CCK-8 assays were performed to evaluate the cellular growth curves in 24h, 48h, 72h (H). (I) ﻿EdU assays were performed to detect cell proliferation in primary HC ﻿and quantitatively compared. The data are expressed as the mean ± SD for at least triplicate experiments. **p*<0.05 stands for vs siRNA-Control or LV-Control.


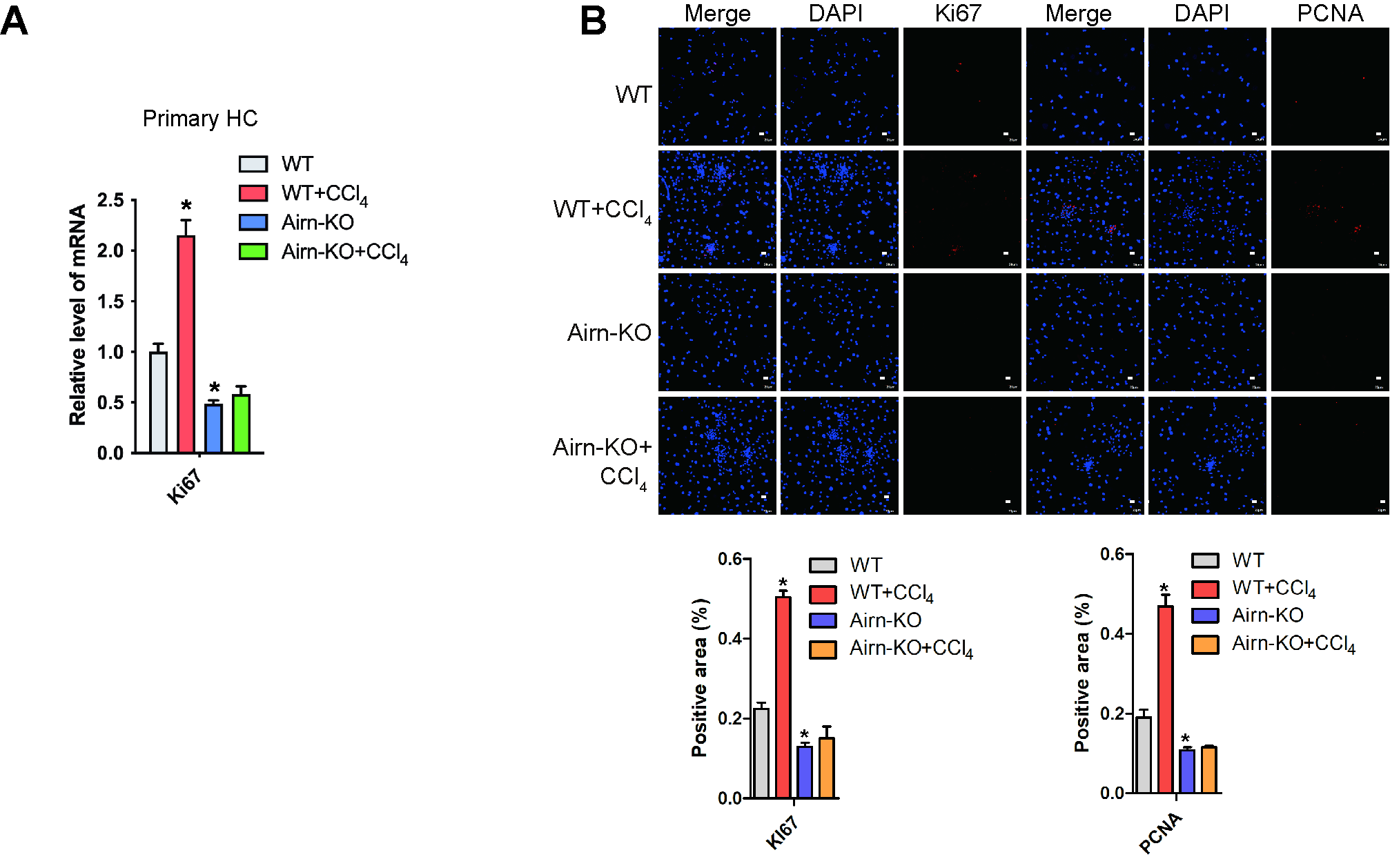


**Fig. S9, related to Fig. 6.** (A-B) Primary HC were isolated from four groups including WT, WT + CCl_4_, *Airn*-KO, *Airn*-KO + CCl_4_. The expression of *Ki67* was detected by qRT-PCR (A). The expression of Ki67 and PCNA was detected by confocal microscopy ﻿and quantitatively compared (B). The data are expressed as the mean ± SD for at least triplicate experiments. **p*<0.05 stands for vs WT.


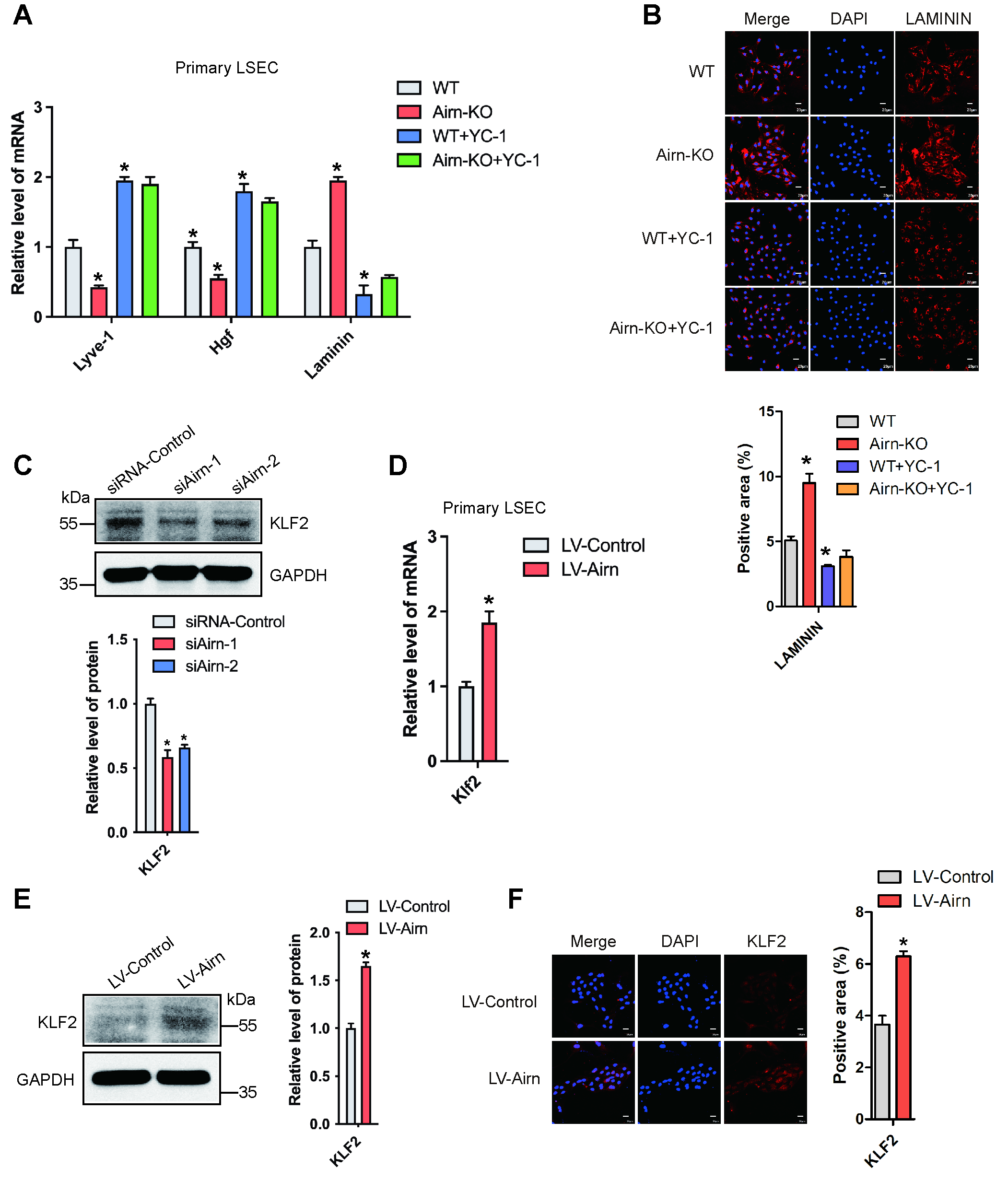


**Fig. S10, related to Fig. 7.** Primary LSEC were isolated from four groups including WT, WT + CCl_4_, *Airn*-KO, *Airn*-KO + CCl_4_ and subsequently treated with YC-1. (A) The mRNA level of *Lyve-1*, *Hgf* and *Laminin* was detected by qRT-PCR. (B) The level of LAMININ was determined by confocal microscopy ﻿and quantitatively compared. DAPI stained nuclei blue; scale bar, 20μm. (C) Primary LSEC were transfected with si*Airn* or siRNA-Control for 48 h, the expression of KLF2 was detected by western blot and quantitatively compared. (D-F) Primary LSEC were transfected with LV-*Airn* or LV-Control for 72 h, the expression of KLF2 was detected by qRT-PCR (D), western blot (E) and confocal microscopy (F) ﻿and quantitatively compared. DAPI-stained nuclei blue; scale bar, 20μm. The data are expressed as the mean ± SD for at least triplicate experiments. **p*<0.05, stands for vs WT or LV-Control.


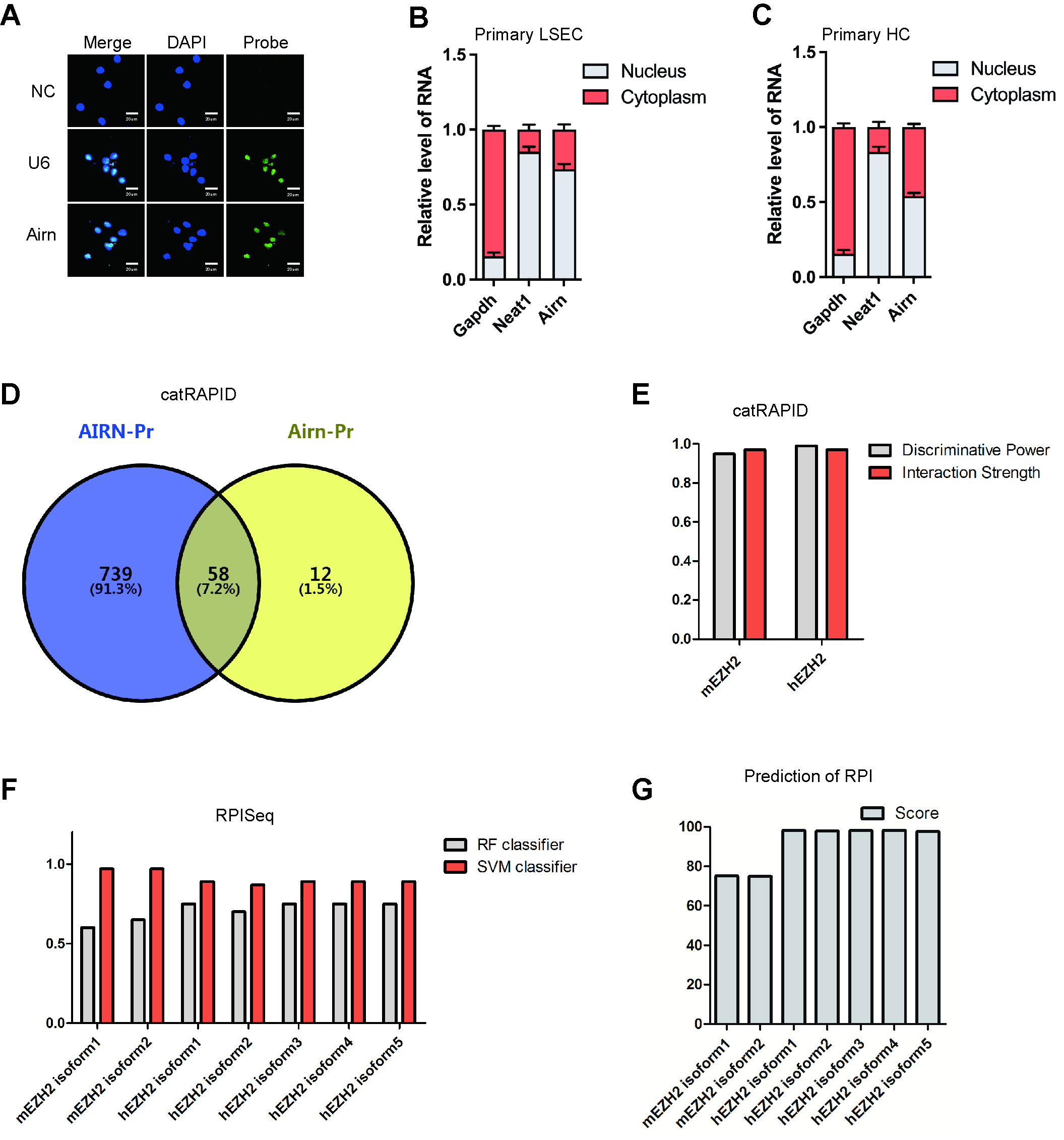


**Fig. S11, related to Fig. 7.** (A) The intracellular localization of *Airn* in LSEC was measured by RNA-FISH assays. U6 was the nuclear control and NC was the negative control, scale bar, 20 μm. (B, C) RNA was extracted from the nuclei or cytoplasm of primary LSEC and HC. qRT-PCR analysis was used to detect *Gapdh* (cytoplasm retained), *Neat1* (nuclears retained) and *Airn* expression. (D) The Venn crosstalk between human *AIRN*-binding proteins and mouse *Airn*-binding proteins predicted by catRAPID database. (E-G) Prediction of the interaction probabilities between *Airn* and EZH2 using the catRAPID (E), RPISeq (F) and prediction of lncRNA-protein interactions database (G). The data are expressed as the mean ± SD for at least triplicate experiments, **p*< 0.05.


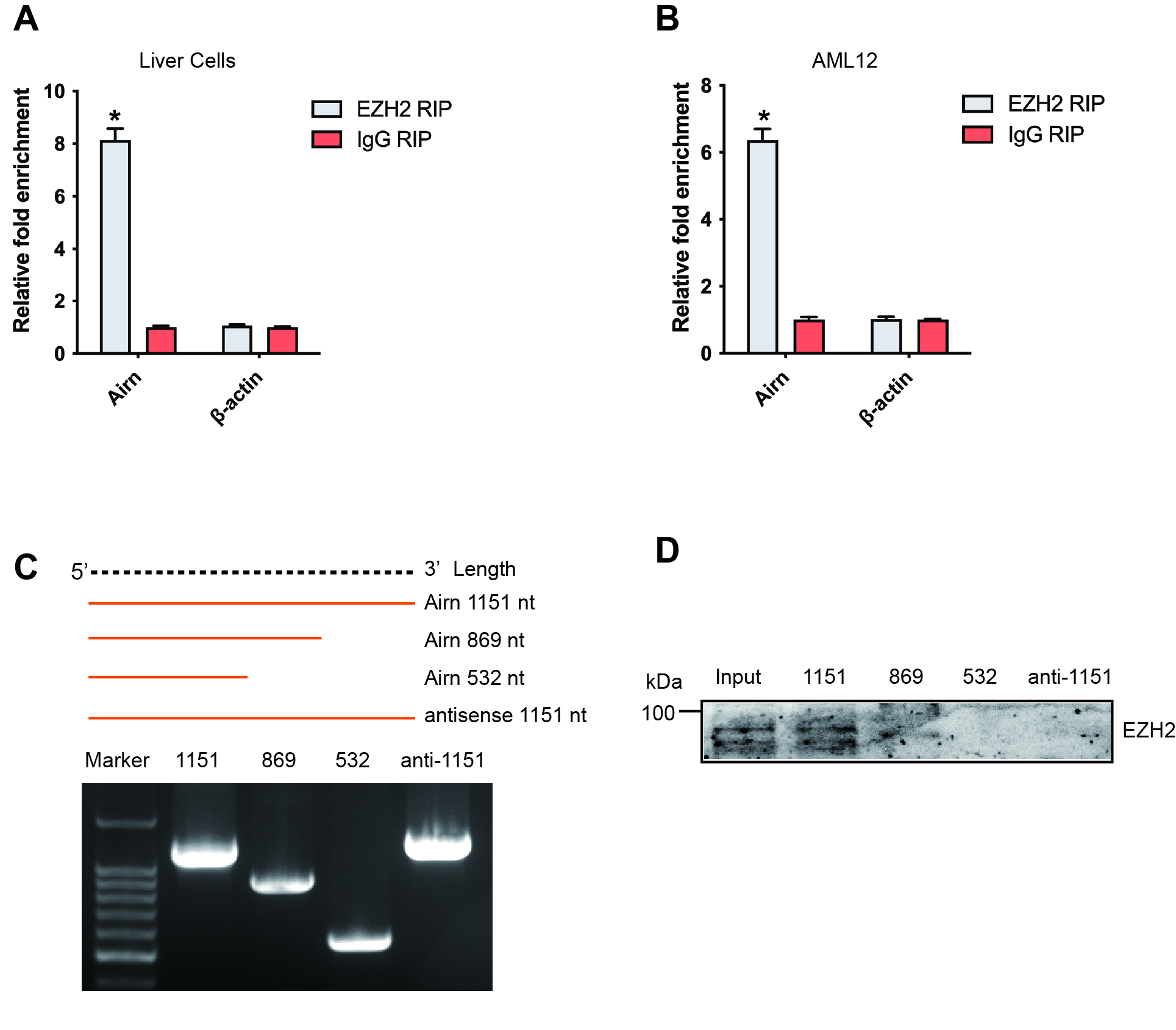


**Fig. S12, related to Fig. 7.** ***Airn* interacted with EZH2.** (A, B) qRT-PCR detection of *Airn* and *β-actin* retrieved by EZH2-specific antibody compared with IgG in the RIP assay with the single cell suspensions isolated from mouse liver (A) and AML12 cells (B). (C, D) RNA Pull-down assay for full-length or truncated *Airn* and the indicated antisense probe (C), followed by western blotting (D) using the EZH2 antibody. The data are expressed as the mean ± SD for at least triplicate experiments, **p*< 0.05.

**
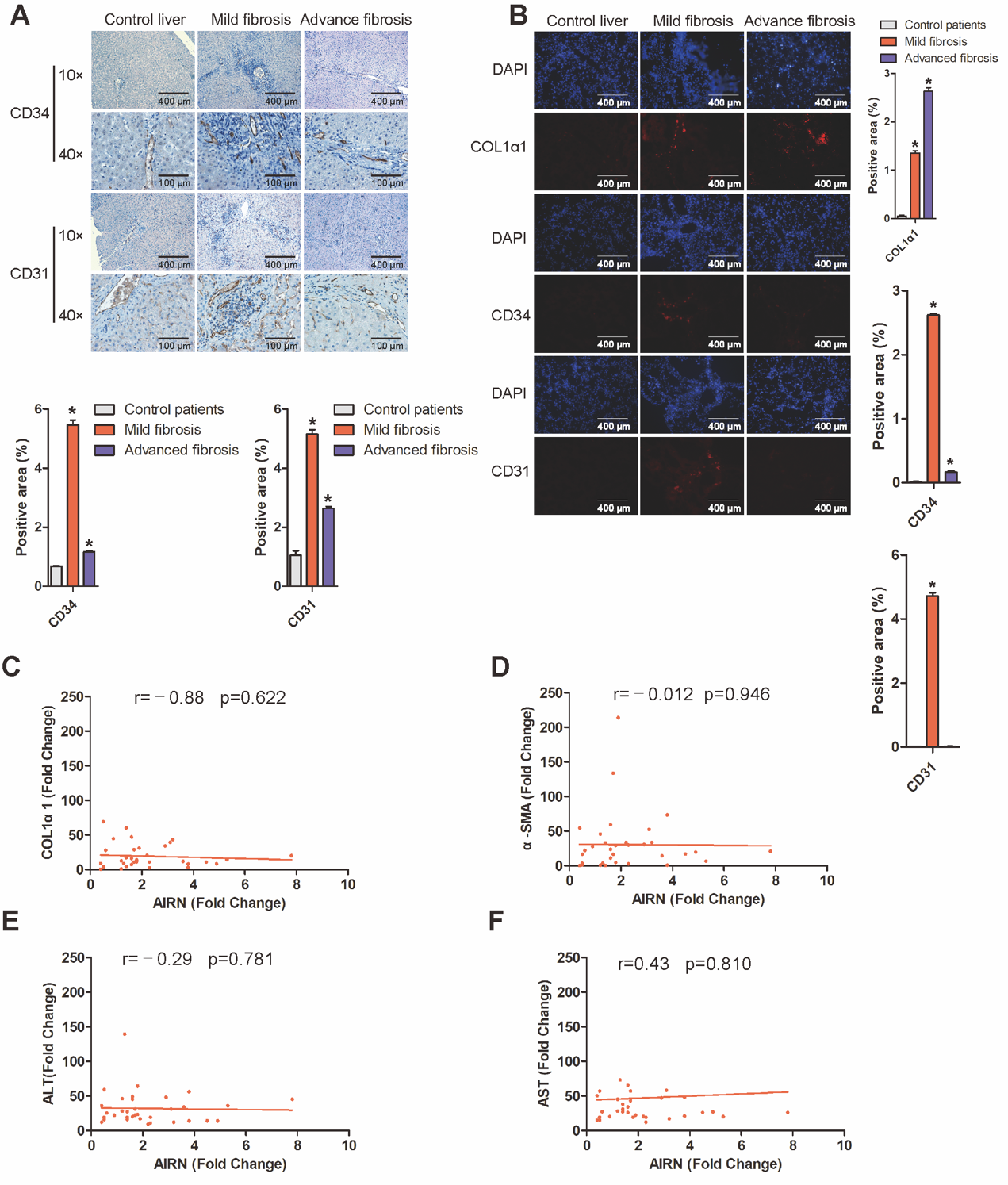
**

**Fig. S13.** **The correlation between *AIRN* level and angiogenesis or fibrosis.** (A) Human liver tissues were divided into Control liver, Mild fibrosis and Advance fibrosis and the expression of CD34 and CD31 was determined by IHC, five images of each liver and five livers from different mice were quantified for each group; scale bar, 400 μm for 10× and 100 μm for 40×. (B) Mouse liver tissues were divided into Control liver, Mild fibrosis and Advance fibrosis and the expression of COL1α1, CD34 and CD31 was determined by immunofluorescence. Right, five images of each liver and five livers from different mice were quantified for each group; DAPI-stained nuclei blue; scale bar, 400μm. (C-F) The correlation between *AIRN* level and COL1α1 (C), α-SMA (D), ALT (E) and AST (F) in patient liver tissues was assessed using Pearson’ correlation analysis, n=34.


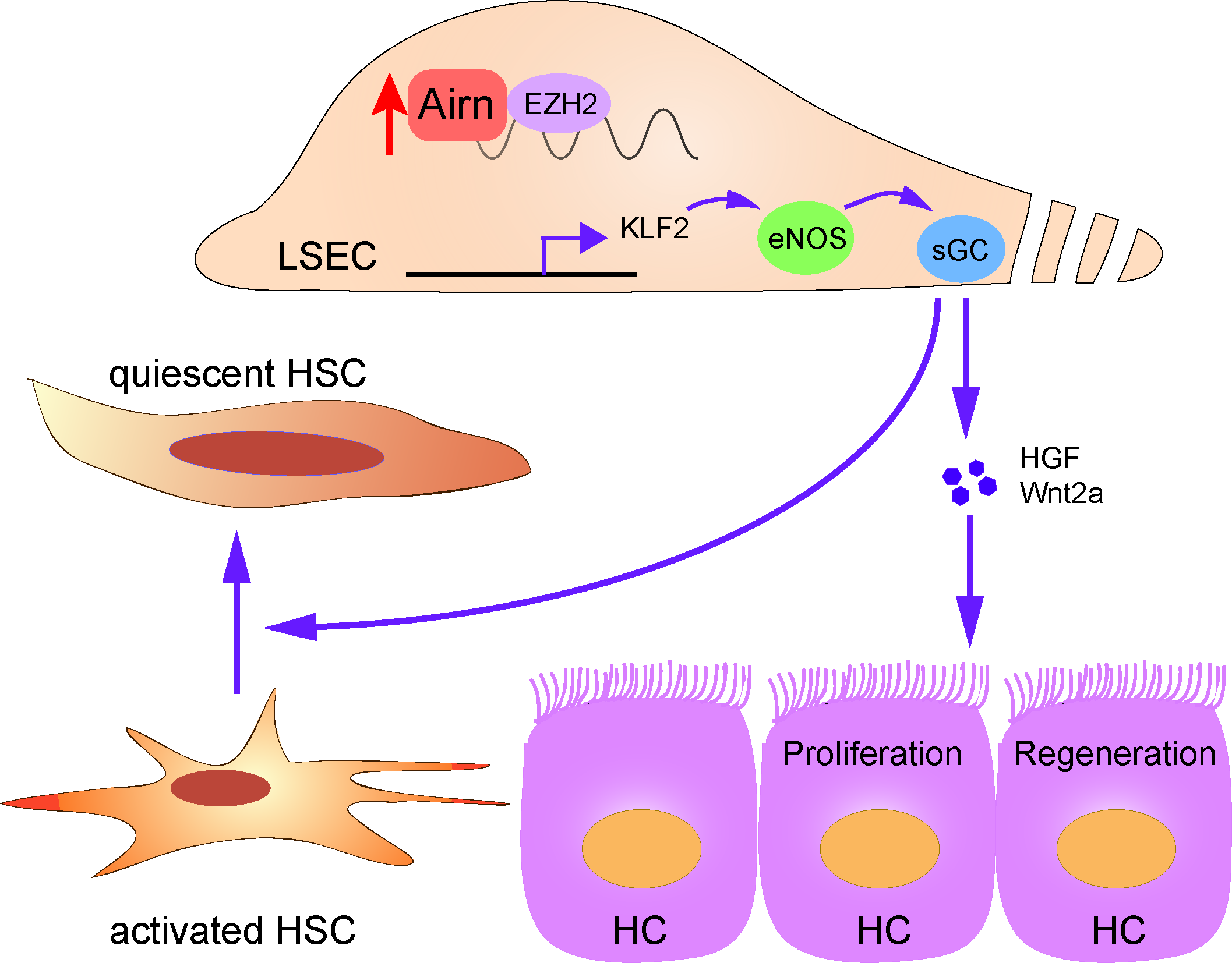


**Fig. S14. Schematic diagram illustrates the role and mechanism of *Airn* in the differentiation of LSEC and liver fibrosis.** *Airn* suppressed the binding of EZH2 to the promoters of KLF2 and the target genes, promoting the expression of KLF2, thereby activating eNOS-sGC pathway, which maintained LSEC differentiation, thereby inhibiting HSC activation indirectly by regulating LSEC differentiation and promoting HC proliferation by the increased paracrine secretion of Wnt2a and HGF from LSEC.

**Supplementary Tables**

**Table S1. Baseline characteristics of patients with fibrotic liver serum**

| Metavir score | Healthy | Mild fibrosis |
| --- | --- | --- |
| Cases(n) | 30 | 47 |
| Age (years)* | 50.2±3.4 | 54.8±1.7 |
| Male sex (n (%)) | 18(60) | 29(61.7) |
| ALT (U/L)* | 20.5±3.1 | 32.0±4.0 |
| AST (U/L)* | 26.0±5.3 | 36.0±4.1 |
| ALB (g/L)* | 42.6±2.8 | 48.0±0.4 |
| GGT (U/L)* | 32±3.9 | 51.3±8.7 |
| Etiology (n (%))  Biliary Obstruction | 0 | 4(8.5) |
| HBV  HCV | 0  0 | 43(91.5)  0 |

*Mean±SD.

ALT, alanine aminotransferase; AST, aspartate aminotransferase; ALB, Albumin; GGT, γ-glutamyl transpeptadase; HBV, hepatitis B virus; HCV, hepatitis C virus.

**Table S2. Serum ALT, AST and liver hydroxyproline levels in CCl_4_-induced liver fibrosis model (mean ± SD, n = 8)**

Group ALT (U/L) AST (U/L) Hydroxyproline (μg/g)

WT group 18.6 ± 2.6 17.3 ± 8.2 164.0 ± 19

WT+CCl_4_ group 135.3 ± 16.5* 93.0 ± 20.0* 277.3 ± 25.1*

*Airn*-KO group 23.3 ± 2.1 21.9 ± 2.1 184.3 ± 8.1

*Airn*-KO+CCl_4_ group 125.3 ± 5.3 151.8 ± 32.9# 395.7 ± 40.5#

**p*<0.05 compared with the WT group. #*p*<0.05 compared with WT + CCl_4_ group. All statistical analyses were performed using SPSS version 13.0 software and *p*<0.05 indicated statistical significance.

**Table S3. Serum ALT, AST and liver hydroxyproline levels in BDL-induced liver fibrosis model (mean ± SD, n = 8)**

Group ALT (U/L) AST (U/L) Hydroxyproline (μg/g)

WT group  19.0 ± 8.0 36.3 ± 18.1 185 ± 51.5

WT+BDL group 221.5 ± 44.9* 207.8 ± 73.9* 309.7 ± 10.0*

*Airn*-KO group 20.7 ± 5.8 26.6 ± 4.9 154.7 ± 24.5

*Airn*-KO+BDL group 358.5 ± 113.5# 770.5 ± 159.9# 431.3 ± 50.9#

**p*<0.05 compared with the WT group. #*p*<0.05 compared with WT + BDL group. All statistical analyses were performed using SPSS version 13.0 software and *p*<0.05 indicated statistical significance.

**Table S4. Serum ALT, AST and liver hydroxyproline levels in CCl_4_-induced liver fibrosis model (mean ± SD, n = 8)**

Group ALT (U/L) AST (U/L) Hydroxyproline (μg/g)

AAV8-GFP group 15.7 ± 2.4 25.9 ± 4.9 149.7 ±31.3

AAV8-GFP+CCl_4_ group 156.9 ± 13.7* 123.8 ± 38.9* 348.7 ± 73.7*

AAV8-*Airn* group 15.8 ± 5.6 24.8 ± 8.3 159.7 ± 33.5

AAV8-*Airn*+CCl_4_ group 99.1 ± 13.3# 128.3 ± 25.7 201.0 ± 11.1#

**p*<0.05 compared with the AAV8-GFP group. #*p*<0.05 compared with AAV8-GFP+CCl_4_ group. All statistical analyses were performed using SPSS version 13.0 software and *p*<0.05 indicated statistical significance.
