# Supplementary figures and images for: LncRNA Airn maintains LSEC differentiation to alleviate liver fibrosis via the KLF2-eNOS-sGC pathway
Source: BMC Med. 2022 Sep 29;20:335. doi: 10.1186/s12916-022-02523-w (PMC9520944; doi:10.1186/s12916-022-02523-w)

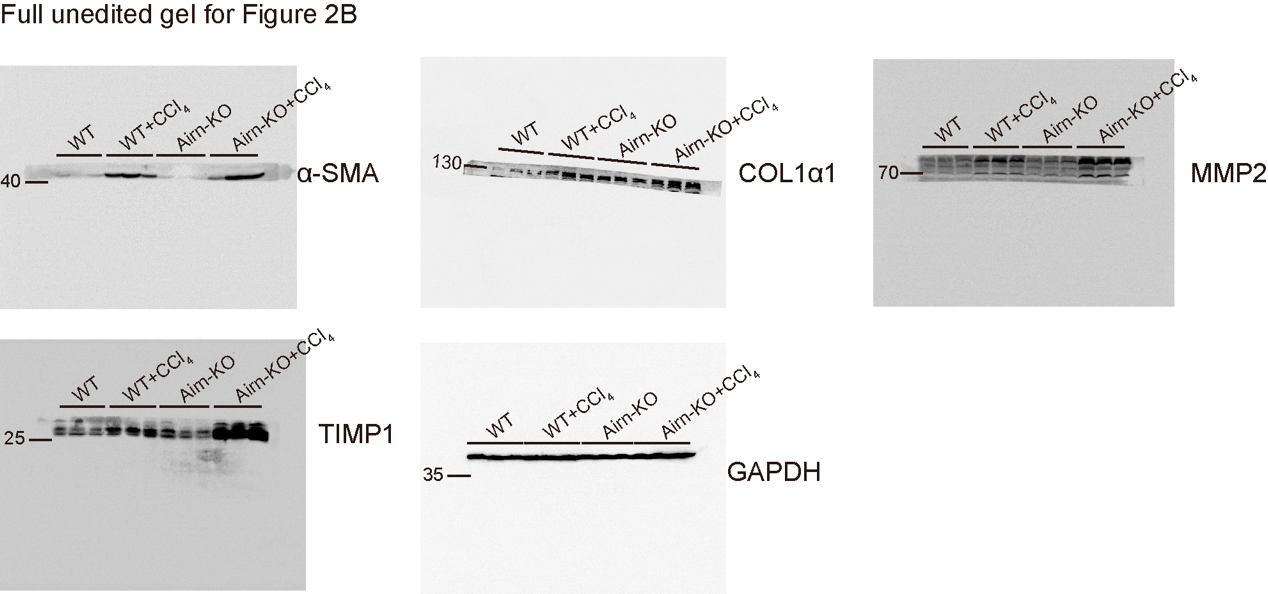


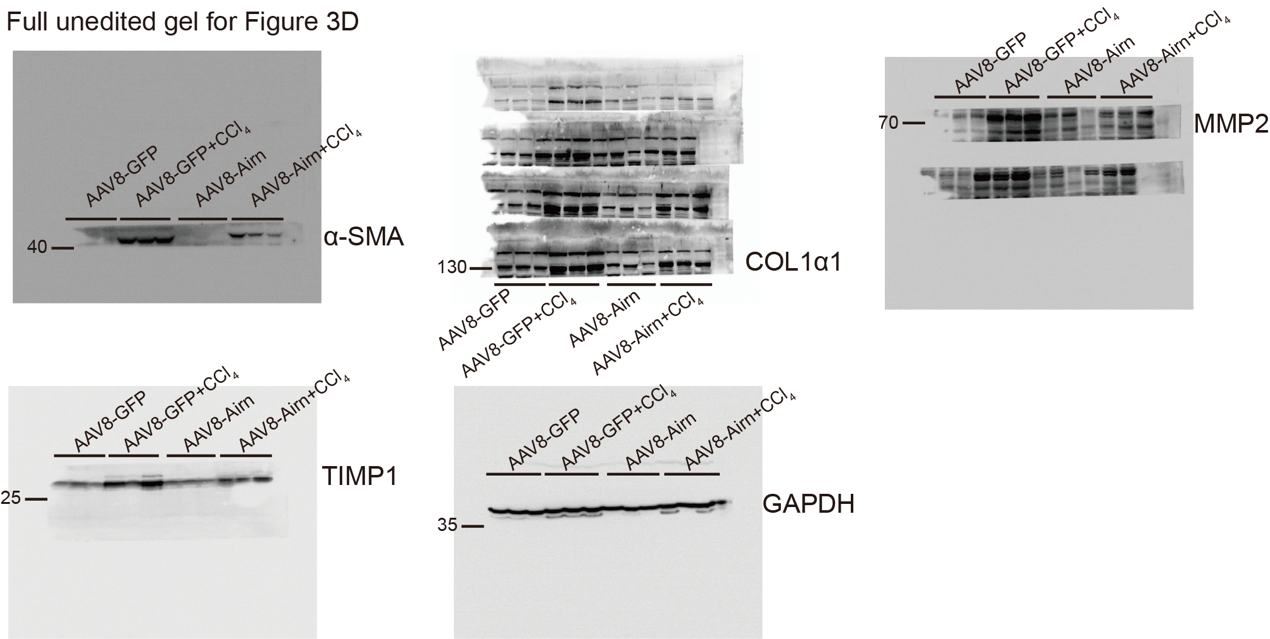


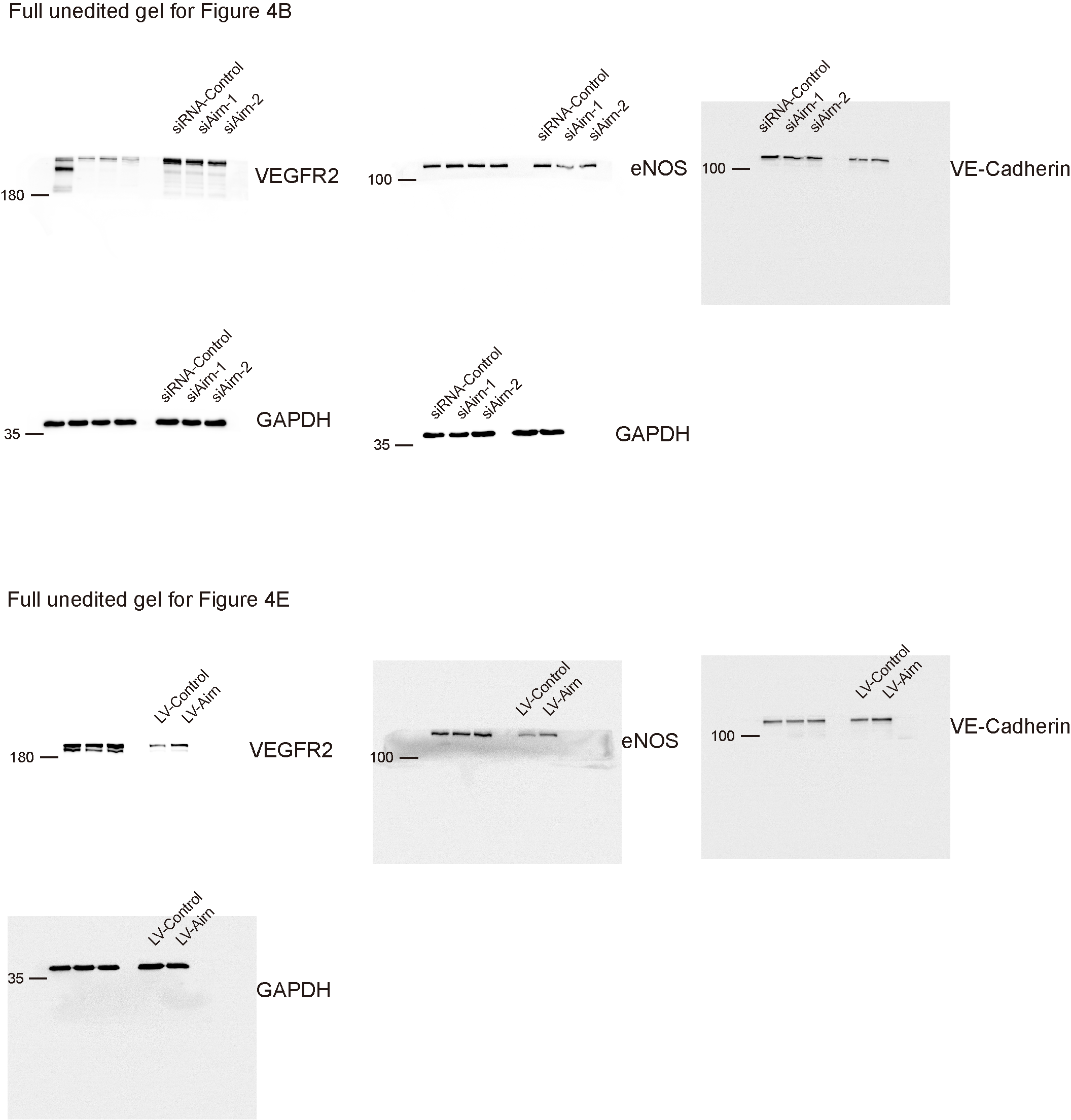


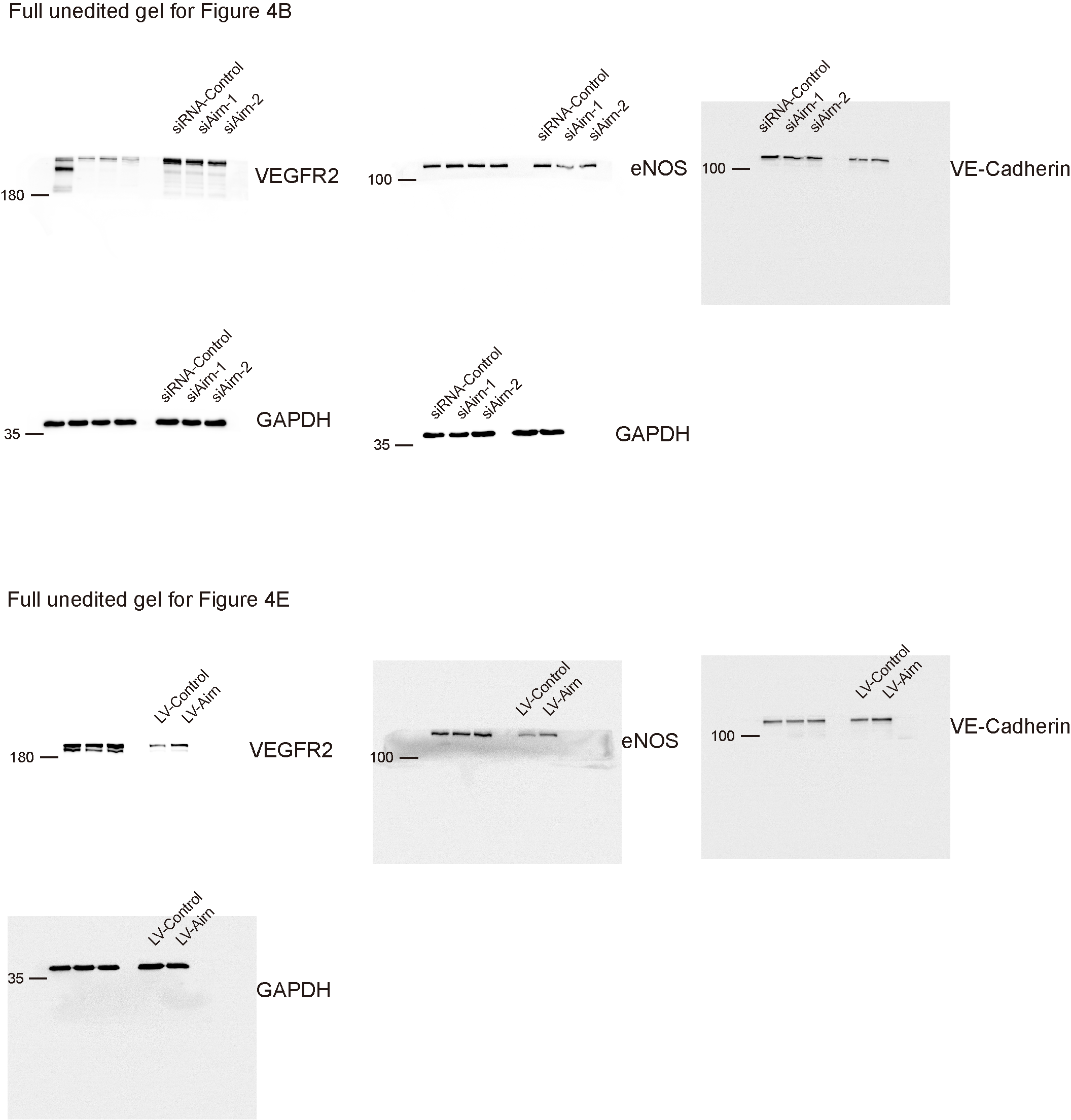


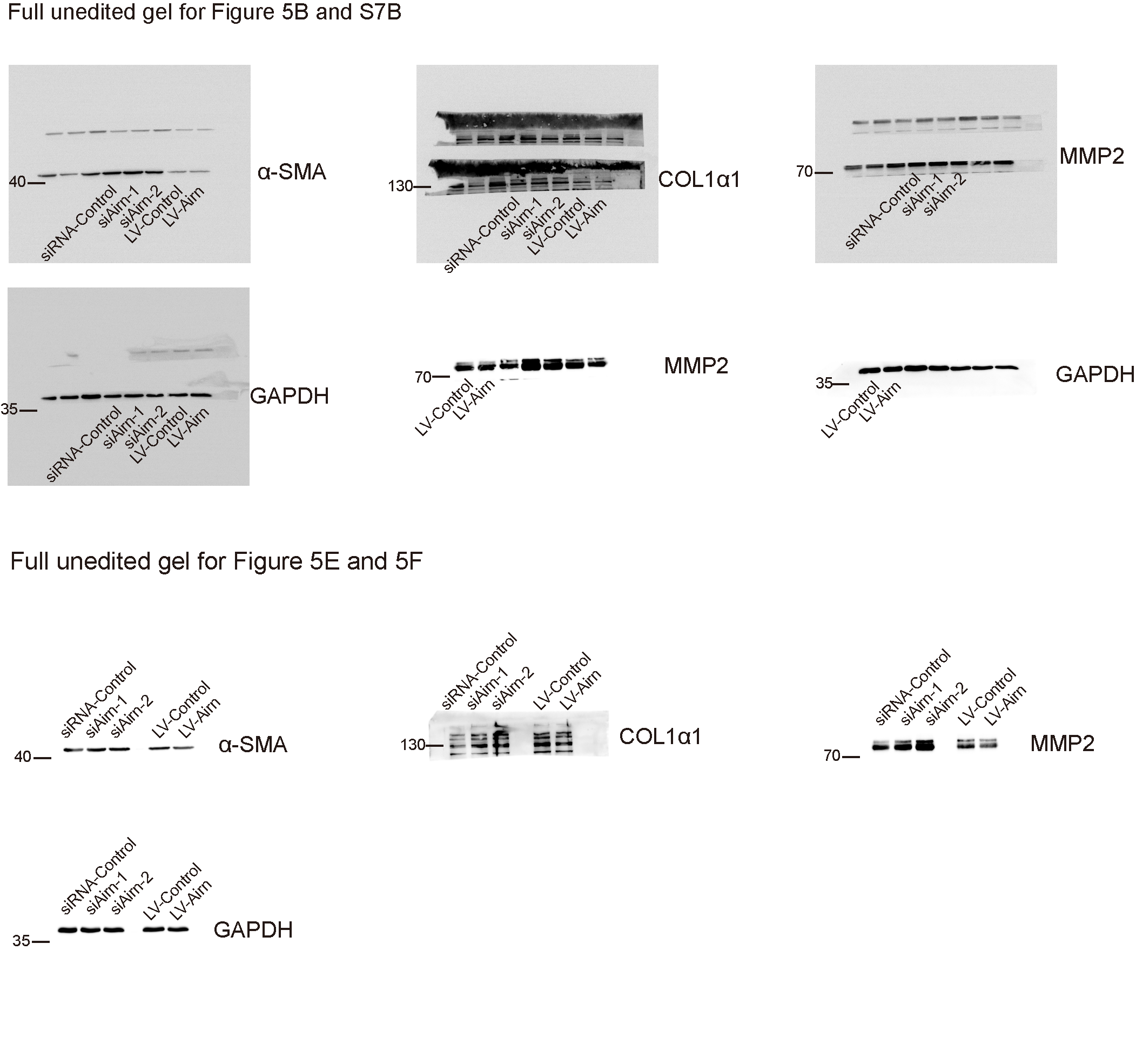


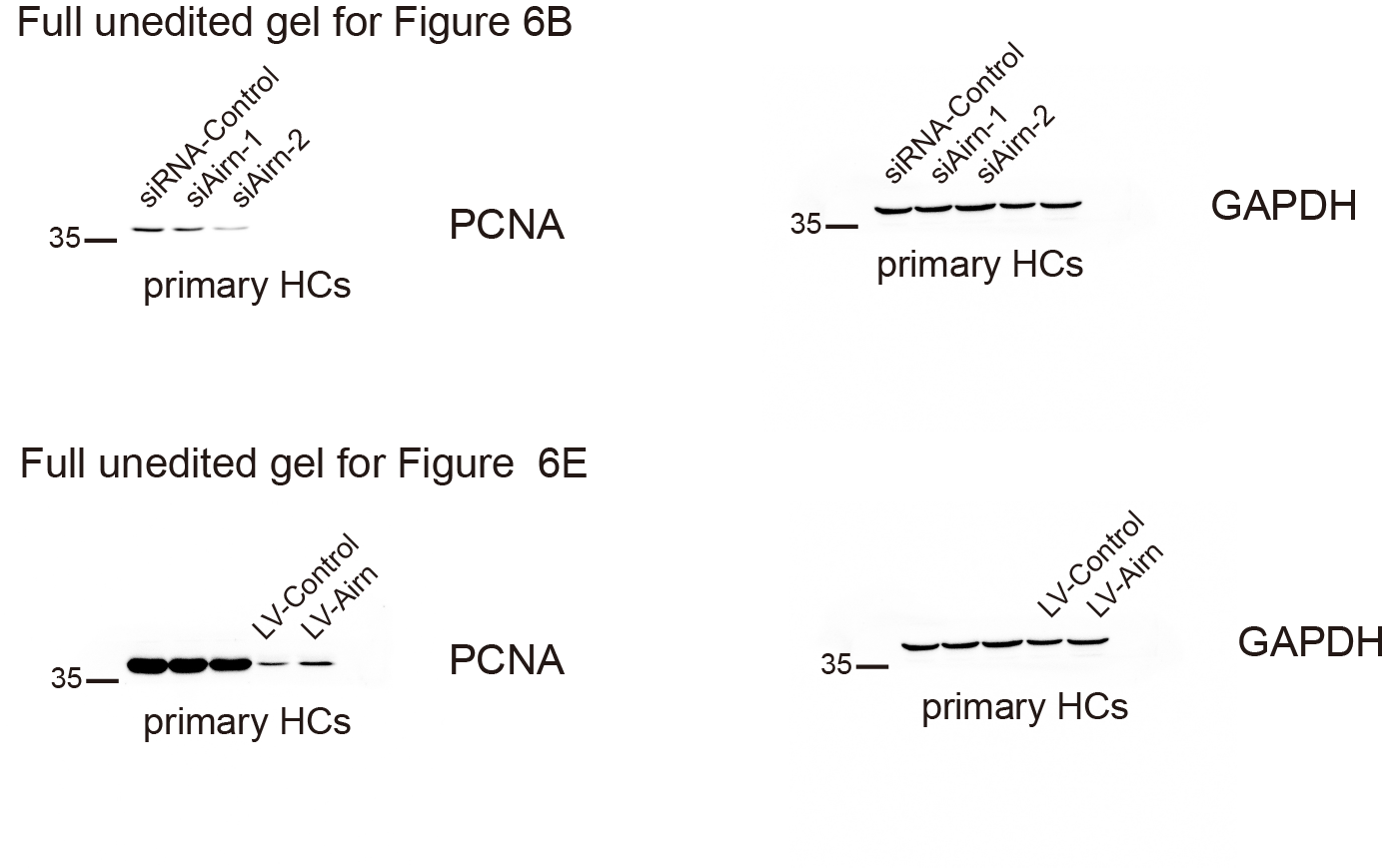


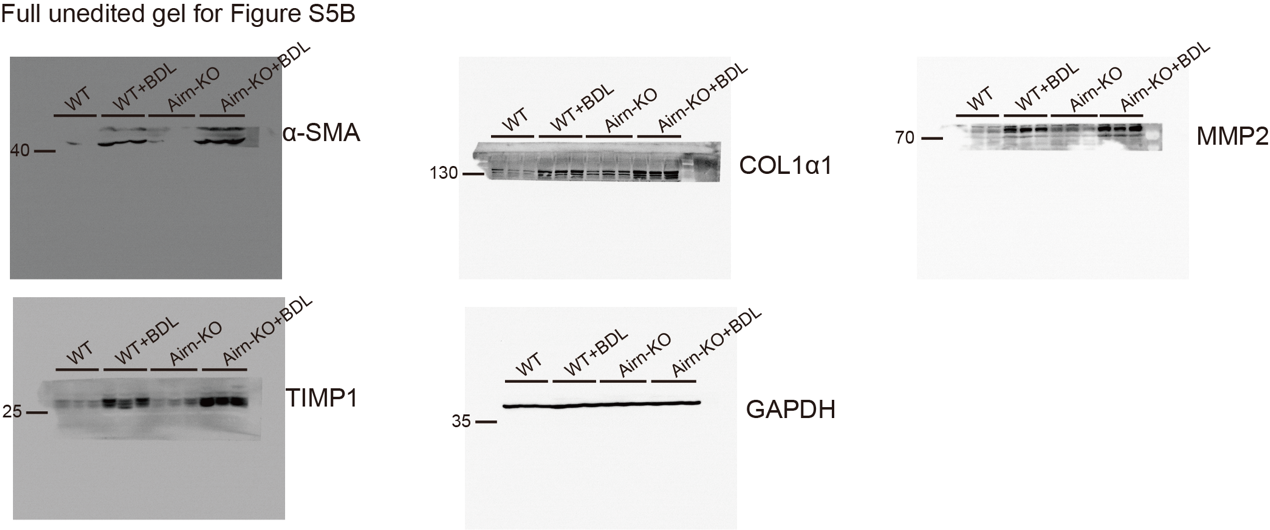


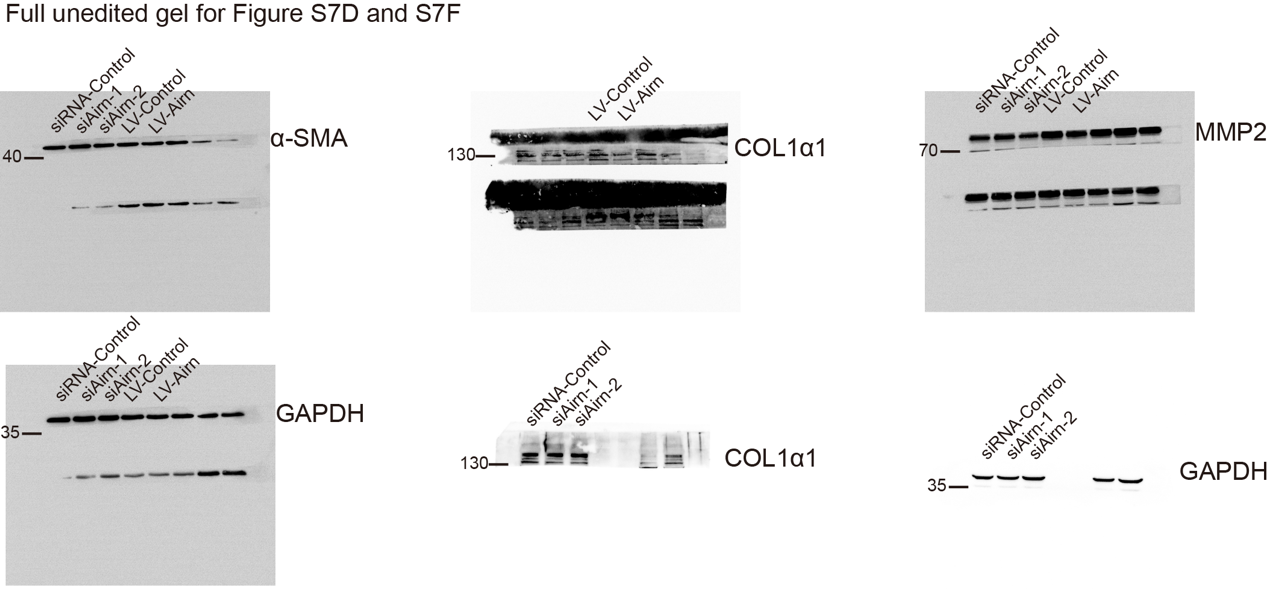


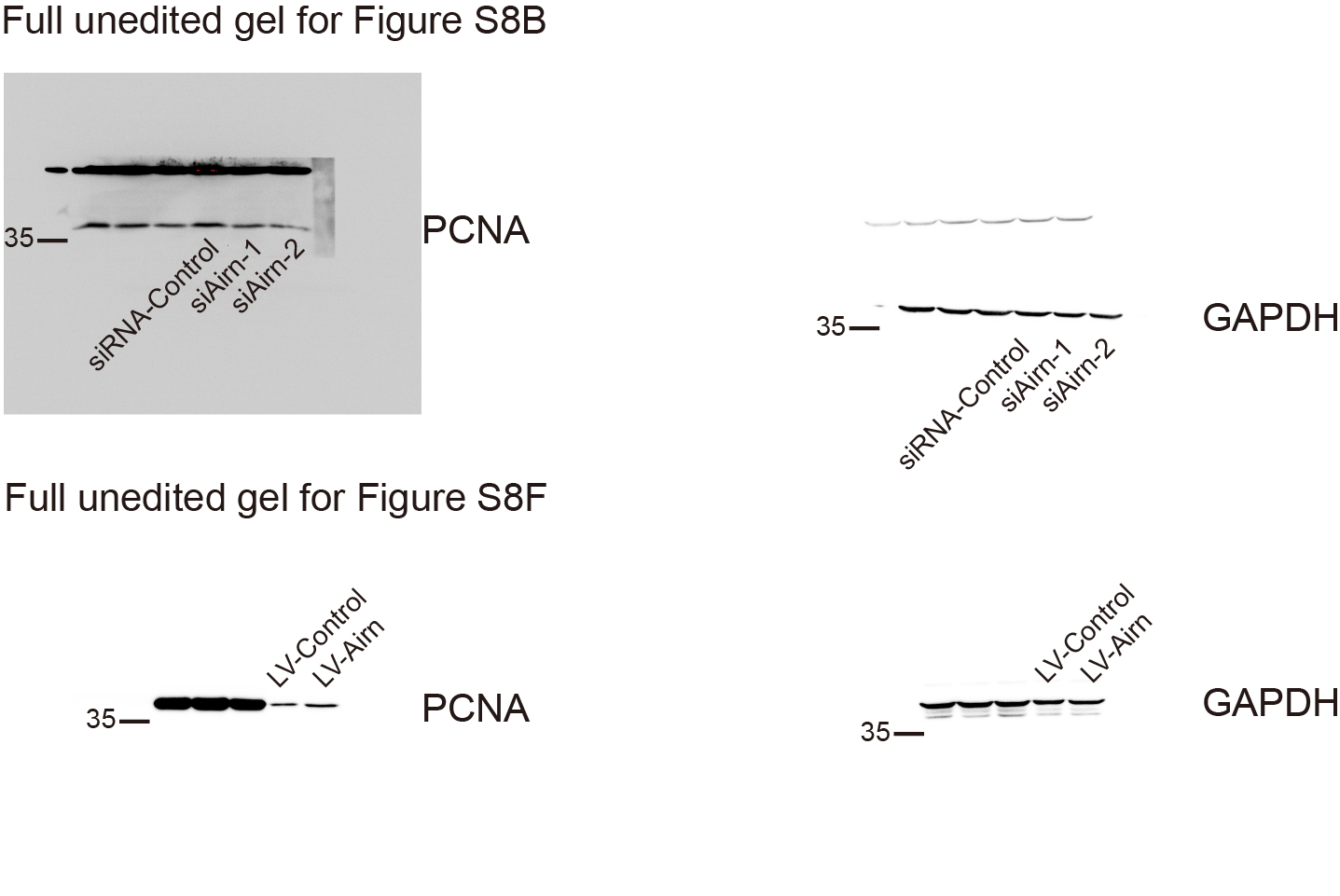


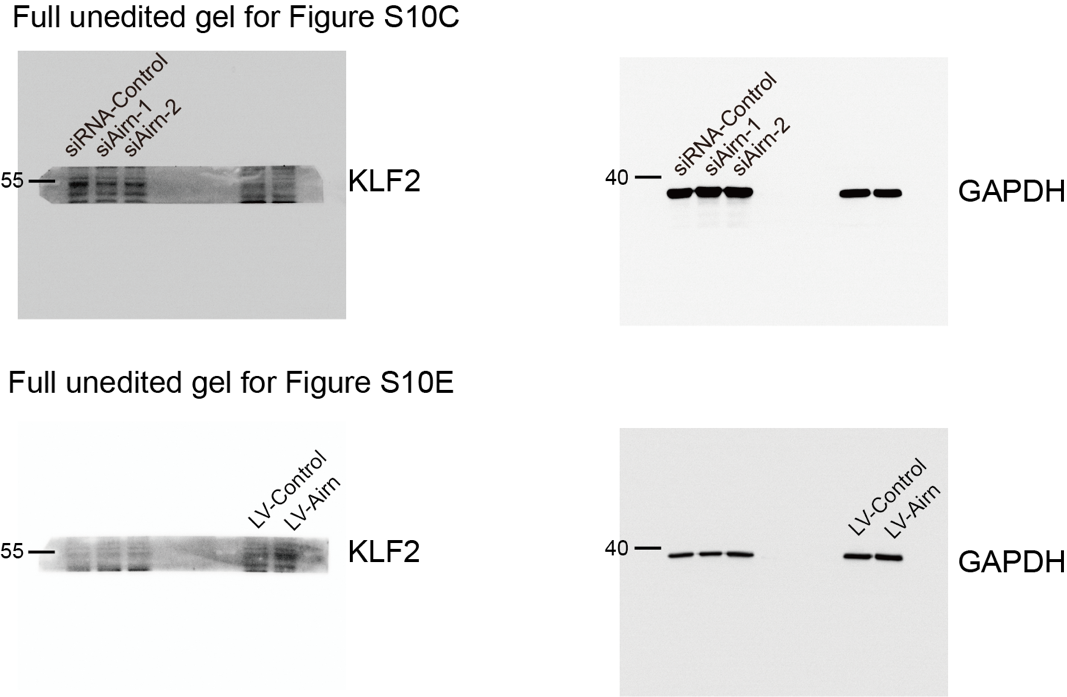


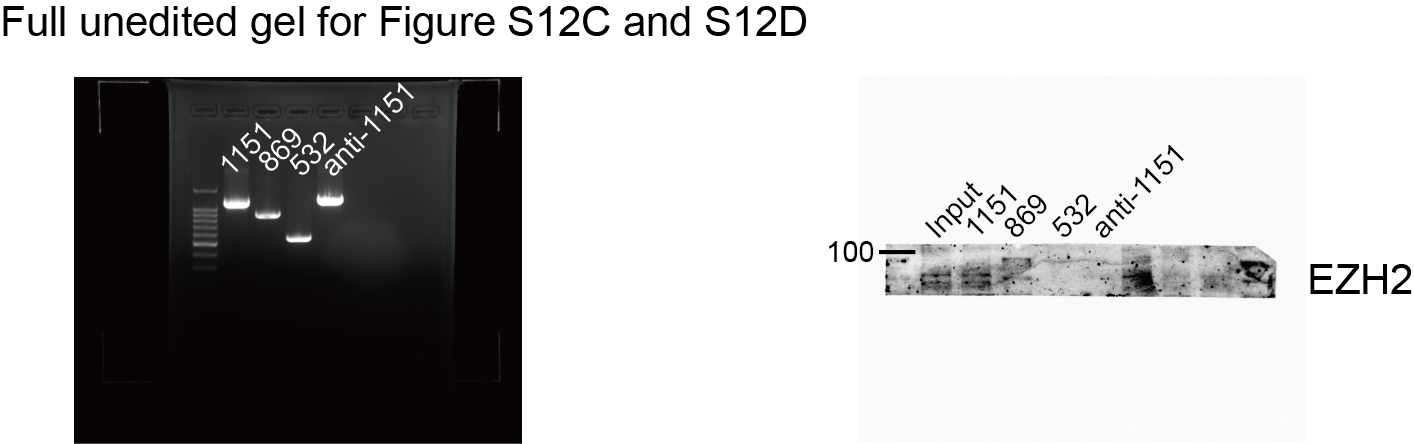

Supplement: Supplementary file 2 — Additional file 2. [file 12916_2022_2523_MOESM2_ESM.docx]
